# Predicting and clustering plant *CLE* genes with a new method developed specifically for short amino acid sequences

**DOI:** 10.1186/s12864-020-07114-8

**Published:** 2020-10-12

**Authors:** Zhe Zhang, Lei Liu, Melis Kucukoglu, Dongdong Tian, Robert M. Larkin, Xueping Shi, Bo Zheng

**Affiliations:** 1 Key Laboratory of Horticultural Plant Biology of Ministry of Education, Huazhong Agricultural University, Wuhan, 430070 China; 2 College of Horticulture and Forestry Sciences, Huazhong Agricultural University, Wuhan, 430070 China; 3 Institute of Biotechnology, Helsinki Institute of Life Science (HILIFE), University of Helsinki, 00014 Helsinki, Finland; 4 Viikki Plant Science Centre, University of Helsinki, 00014 Helsinki, Finland

**Keywords:** Peptide hormone, CLE, Machine learning, Euclidean distance, Gene prediction, Gene clustering, Evolution

## Abstract

**Background:**

The *CLV3*/*ESR-RELATED* (*CLE*) gene family encodes small secreted peptides (SSPs) and plays vital roles in plant growth and development by promoting cell-to-cell communication. The prediction and classification of *CLE* genes is challenging because of their low sequence similarity.

**Results:**

We developed a machine learning-aided method for predicting *CLE* genes by using a CLE motif-specific residual score matrix and a novel clustering method based on the Euclidean distance of 12 amino acid residues from the CLE motif in a site-weight dependent manner. In total, 2156 CLE candidates—including 627 novel candidates—were predicted from 69 plant species. The results from our CLE motif-based clustering are consistent with previous reports using the entire pre-propeptide. Characterization of CLE candidates provided systematic statistics on protein lengths, signal peptides, relative motif positions, amino acid compositions of different parts of the CLE precursor proteins, and decisive factors of CLE prediction. The approach taken here provides information on the evolution of the *CLE* gene family and provides evidence that the *CLE* and *IDA/IDL* genes share a common ancestor.

**Conclusions:**

Our new approach is applicable to SSPs or other proteins with short conserved domains and hence, provides a useful tool for gene prediction, classification and evolutionary analysis.

## Background

Small secreted peptides (SSPs) play vital roles in cell-to-cell communication during plant growth and development [1–4]. The most well understood plant SSPs are encoded by the *CLAVATA3* (*CLV3*)/*EMBRYO SURROUNDING REGION* (*ESR*)-*RELATED* (*CLE*) gene family [5, 6]. CLE peptides have been widely identified in bryophytes, pteridophytes, gymnosperms and angiosperms [7]. A typical CLE protein contains an N-terminal signal peptide, a non-conserved variable region in the middle, a C-terminal conserved motif (CLE motif) and in some instances, a short C-terminal tail downstream of the CLE motif. CLE motifs are usually composed of 12 to 13 amino acid residues. Exogenous peptides containing the CLE motif can mimic the phenotypes of transgenic plants that overexpress *CLE* genes [8–10]. The conserved CLE domains contain hydroxyproline and arabinosylated hydroxyproline residues [11–13]. Interestingly, the influence of these post-translational modifications varies in different species. For instance, post-translational modifications are critical for the activity of the CLV3 peptides in tomato but not in *Arabidopsis* [14, 15]. Typically, the mature forms of CLE peptides are recognized by plasma membrane-localized leucine-rich repeat receptor-like kinases (LRR-RLK) or receptor-like proteins (LRR-RLP) [16–18]. The extracellular domains of LRR-RLK/RLPs bind CLE peptides. Ligand binding activates the intracellular domain of the LRR-RLKs or plasma membrane-associated kinases and thus, transduces the extracellular signal. Various methods have been used to investigate the interactions between CLE peptides and LRR-RLK/RLPs, such as genetic and physiological approaches, directly quantifying physical interactions and phosphor-proteomics. However, only a small number of possible ligand-receptor pairs have been identified [19, 20].

CLE peptides regulate the growth and development of various tissues in *Arabidopsis*, such as the apical and vascular meristems. Well-known CLE peptides include CLV3*,* CLE40 and TRACHEARY ELEMENT DIFFERENTIATION INHIBITORY FACTOR (TDIF), which regulate the *CLAVATA1* (*CLV1*)–*WUSCHEL* (*WUS*) signaling pathway in the shoot apical meristem (SAM) [21], the *ARABIDOPSIS CRINKLY4* (*ACR4*)–*WUSCHEL*-related *Homeobox5* (*WOX5*) signaling pathway in the root apical meristem (RAM) [22], and the *TDIF RECEPTOR*/*PHLOEM INTERCALATED WITH XYLEM* (*TDR*/*PXY*)–*WOX4* signaling pathway in the vascular cambial meristem (CAM) [10, 17, 23], respectively. In addition, *CLE6* promotes gibberellin (GA)-mediated shoot growth [24]. The *CLE8*–*WOX8* signaling pathway regulates endosperm and embryo development [25]. CLE9 and CLE10 regulate the formation of protoxylem by binding BARELY ANY MERISTEM (BAM) [26, 27]. CLE9/CLE10–HAESA-LIKE1 (HSL1)-SOMATIC EMBRYOGENESIS RECEPTOR KINASEs (SERKs) regulate stomatal lineage cell division [27]. *CLE20* inhibits root growth and lateral root growth by inhibiting RAM and CAM activity, respectively [28, 29]. *CLE25*–*BAM* induces stomatal closure and promotes drought tolerance by controlling abscisic acid accumulation. Additionally, *CLE25* promotes phloem initiation by regulating a CLE-RESISTANT RECEPTOR KINASE (CLERK)–CLV2 receptor complex [30, 31]. *CLE26* regulates root architecture and protophloem formation [32–34]. *CLE45–BAM3* suppresses protophloem differentiation and RAM growth. In contrast, *CLE45–STERILITY-REGULATING KINASE MEMBER1*/*2* (*SKM1*/*SKM2*) promotes seed production in plants grown in elevated temperatures by maintaining pollen performance [35, 36].

Each amino acid of the CLE motif plays different roles in peptide modification, peptide activity, and ligand–receptor binding [37–41]. For example, *clv3* mutants and plants expressing a CLV3 motif with the Gly residue at the 6th position substituted with Leu, Ile, Val, Phe, Tyr, or Pro are phenotypically similar [38]. Similarly, structural and functional analyses of the TDIF–TDR/PXY (ligand–receptor pair) demonstrate that each amino acid residue of the TDIF motif is important. Indeed, amino acid substitutions at the 1st, 3rd, 4th, 6th, 8th, 9th and 12th positions of the TDIF motif result in reduced or complete loss of function [39]. Although amino acid substitutions at the 2nd, 5th, 10th and 11th sites of the TDIF motif have very little impact on its TE differentiation activity, these sites are important for specifically binding the TDR/PXY receptor [40, 41].

Because of the short coding sequences and the generally low sequence similarity among the CLE proteins, the identification and classification of *CLE* genes has always been a challenge, even in the model plant *A. thaliana*. Originally, using TBLASTN, 39 typical CLE polypeptides were identified. Twenty four of them were from *A. thaliana* [42]. Subsequently, *CLE40*, *CLE41* and a nematode *CLE* (*HgCLE*) gene were identified using the same approach [43]. The latter one provided evidence of ligand mimicry. *CLE41* was the first of a novel class of *CLE* genes, the *TDIF* and *TDIF*-like genes [39, 44]. A total of 32 *CLE* genes have been identified in *A. thaliana* defining 26 unique CLE peptides [45]. The Arabidopsis *CLE* genes have been used to identify *CLE* homologues in many other plant species, such as *Oryza sativa* [46], *Lotus japonicas* [47], *Selaginella moellendorfii* [48], *Medicago truncatula* [49, 50], *Picea abies* [51], *Solanum lycopersicum* [52], *Glycine max* [53], *Raphanus sativus* [54], and *Populus trichocarpa* [55]. Goad et al. [7] predicted CLE polypeptides from 57 plant species. The classification of *CLE* gene families has been based on their functions or sequence similarities. Based on the effects of CLE peptides on plant growth, 22 *Arabidopsis* CLE polypeptides were classified into two groups [56]. According to their physiological functions, four classes of CLE peptides were proposed [57]. On the other hand, 13 categories of CLE motifs were generated by clustering the conserved sequences using the CLANS software [7, 58].

The objective of this study was to develop a novel approach for efficiently and accurately predicting and classifying CLE proteins. The general substitution matrix was replaced with a modified amino acid substitution matrix that is based on the weight of each position of the CLE motif. Machine learning (ML) was used to improve the accuracy of *CLE* gene predictions. This study helps to define the characteristics of different groups of *CLE* genes and therefore, to explore the origin and evolution of the *CLE* gene family.

## Results

### Developing a new residual score matrix for CLE motifs

To predict *CLE* genes in plants, we developed a new residual score matrix for CLE motifs by integrating the amino acid substitution matrix, amino acid usage frequency matrix and site weights of the CLE motif.

**Fig. 1:**
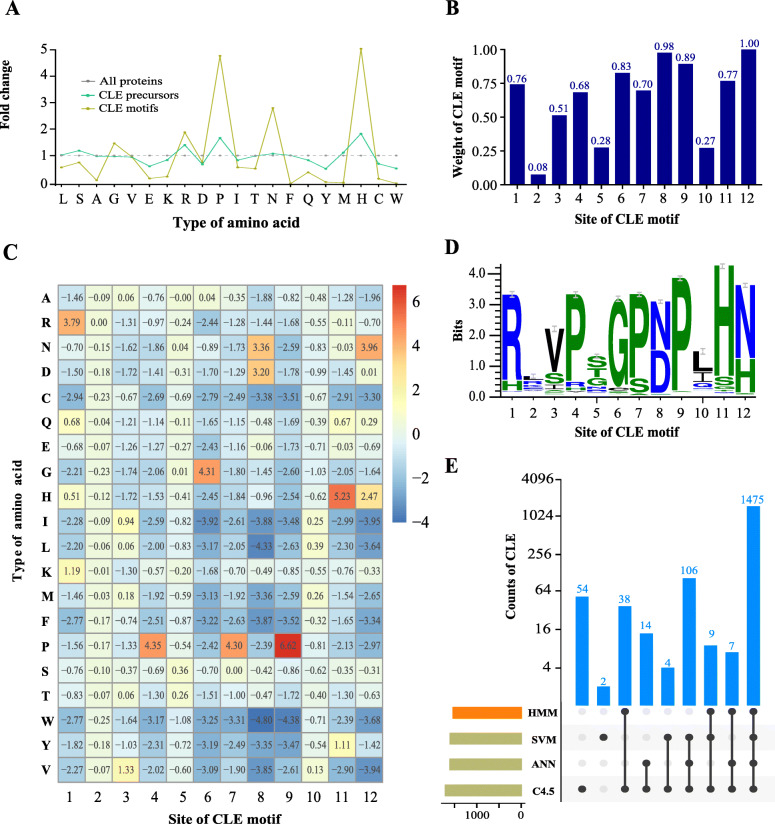
Methods and results for predicting *CLE* genes. **a** Fold changes in the amino acid frequencies in CLE precursors and CLE motifs from 69 species. The amino acid composition of all proteins was used as a control (set to 1.0). The grey, aquamarine and lemon colored lines indicate all proteins, CLE precursors and CLE motifs, respectively. **b** Weight at each site of the CLE motif. **c** Score matrix of CLE motifs. The amino acids are indicated at the left using single letter codes. The numbers in the grid represent the score of each amino acid at sites 1 through 12. **d** Weblogo of the 12-residue CLE motif from the 1529 reported *CLE* genes [7]. **e** UpSet plot for visualizing the intersecting sets of *CLE* genes predicted by different methods. The number of *CLE* genes at each intersection was labeled in blue on the top of the appropriate column

The amino acid composition of the CLE motif was analyzed in 69 species at different levels that included total proteins, small proteins (≤ 200 residues in length) and CLE precursor proteins (Additional file 1: Figure S1). The amino acid composition of small proteins was similar to total proteins (Pearson Correlation Coefficient: 0.23, *p* = 4.4e-17). However, a higher frequency of particular amino acid residues was observed in CLE precursors. For example, the frequency of histidine (H) was 74% higher in CLE precursors than in total proteins (Additional file 1: Figure S1). The amino acid composition in different regions of CLE proteins was also analyzed (Additional file 1: Figure S1). The frequency of P and H in CLE motifs were both more than fourfold higher than in total proteins, which provides evidence that they are functionally important amino acids for peptide processing or for the recognition of peptides by receptors (Fig. 1a). In contrast, some residues were very scarce in CLE motifs, such as the three aromatic amino acids (phenylalanine (F), tyrosine (Y) and tryptophan (W)) and the two sulfur-containing amino acids (cysteine (C) and methionine (M)) (Fig. 1a; Additional file 1: Figure S1). This strong bias in amino acid composition encouraged us to try and build a CLE-specific score matrix.

We tested three commonly used substitution matrices—BLOSUM62, BLOSUM80 and PAM250—and found that the performance of each was similar (Additional file 2: Figure S2). Nevertheless, the BLOSUM80 matrix provided a slightly better resolution of the motif scores and therefore, was used to develop the new score matrix. For the amino acid usage frequency matrix, 1628 reported *CLE* genes from 57 species [7] were chosen as references (Additional file 12: Table S1). The amino acid usage frequency at each site of the CLE motif was calculated as a percentage (Additional file 3: Figure S3) and was represented as a Weblogo sequence (Fig. 1d). The conservativities of each site were largely different, as previously reported [38]. Sites with higher conservativity were considered to hold higher weight in our new score matrix. Some sites contained two dominant residues, such as the 8th site (50.03% N and 47.06% D) and the 12th site (66.49% N and 31.38% H). Based on the modified method for evaluating site weight, the weight of the 12th site was set at 1.00, and the 1st, 6th–9th, and 11th sites had weights no less than 0.70 (Fig. 1b).

In the new CLE score matrix, each residue of a candidate CLE motif made a contribution to its total score. Residues at the conserved sites contributed more than those at less conserved sites. Dominant residues contributed more than scarce residues. For example, the proline at the 9th site alone had a score of 6.62, which made the most striking contribution to the score matrix (Fig. 1a). It is worth mentioning that the combination of 12 residues with the highest frequency at each site was “RLVPSGPNPLHN”, found in CLE9/10 in *A. thaliana*. The combination of residues with the highest score was “RRVPSGPNPLHN”. The total motif score was 38.00.

### Machine learning aided the prediction of *CLE* genes in plants

In addition to the CLE motif score, we also included the protein length, motif position and signal peptide score to predict *CLE* genes in 69 plant species. Three machine learning algorithms, C4.5, ANN and SVM, were employed to categorize all candidate genes into *CLE* genes or non-*CLE* genes using the reported *CLE* genes in the training data set. Our analysis of the training data set, based on 53 species, yielded 1709 *CLE* candidate genes, including the 1529 genes that were predicted using the Hidden Markov Model (HMM) [7] and 180 novel genes. All three machine learning algorithms supported 1475 (96.5%) of the reported *CLE*s and 106 (58.9%) of the novel *CLE*s. In total, 94 (5.5%) of the candidate genes were supported by only one algorithm (Fig. 1e). Additionally, machine learning aided in the prediction of 447 novel *CLE* candidates from the 16 species in the testing data set. Therefore, our method identified a total of 2156 *CLE* candidates in 69 species (Additional file 12: Table S1).

### A new CLE classification method based on the Euclidean distance of CLE motifs in a site-weight dependent manner

**Fig. 2:**
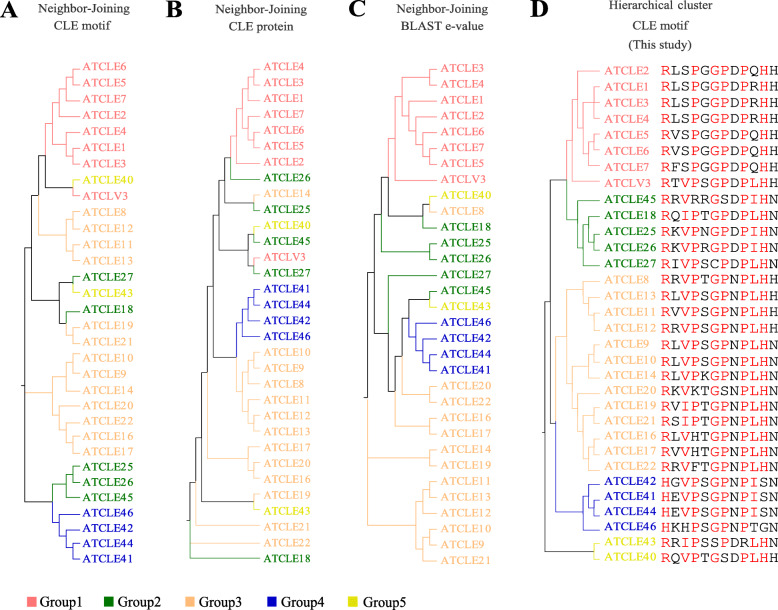
Clustering analysis of Arabidopsis CLE motifs. Phylogenetic tree of AtCLE motifs (**a**), full-length proteins without signal peptides (**b**) and log-normalized rank of all-vs-all BLAST e-values generated using the NJ method based on the evolutionary distances (**c**), which were computed using the Poisson correction method (**a**, **b**), and Euclidean distances (**c**). **d** Clustering of the AtCLE motifs based on the Euclidean distance of each pair of sequences in a site-weight dependent manner. The tree was constructed using the HCL method. The names of the CLE motifs are indicated with different colors

**Fig. 3:**
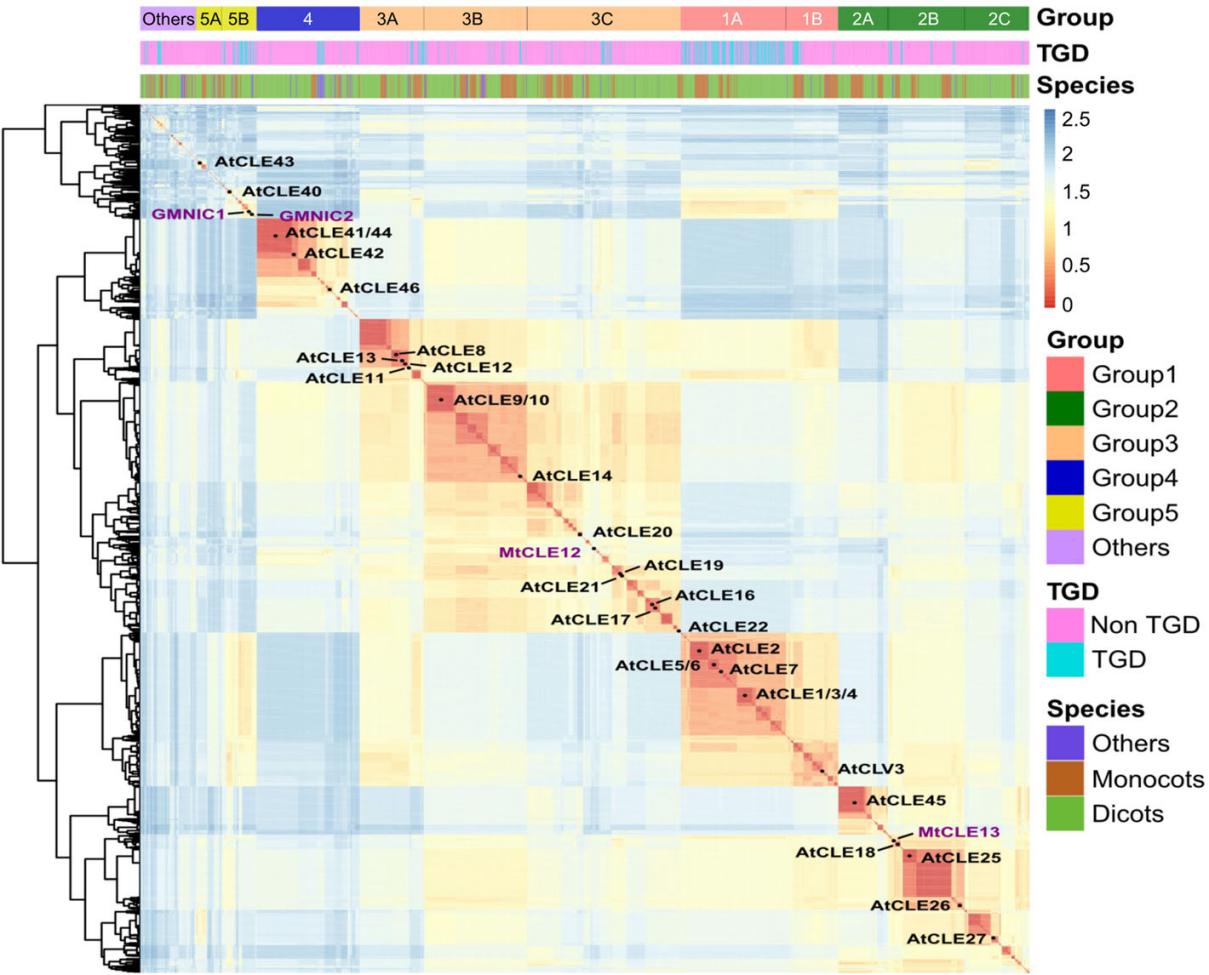
Clustering analysis of CLE motifs in plants. The heat map shows the Euclidean distance of 2156 CLE motifs in 69 plant species. Red represents short distances. Blue represents long distances. A shorter Euclidean distance implies a higher degree of motif similarity. CLE motifs were clustered based on the Euclidean distance of each pair of sequences in a site-weight dependent manner. The clustering tree was generated using the HCL method. The information on the classification of the CLE motifs is shown on the top of the heatmap. All CLE motifs were clustered into six major groups: Group 1–5 and Group “others”. “TGD” and “Non-TGD” indicate whether the motif was from a potential tandem gene duplication (TGD). “Species” indicates that a motif was from a dicot, monocot or other type of plant species

To group the 2156 CLE motifs, the Euclidean distances (*d*) between each CLE candidate and the 32 *Arabidopsis* CLE motifs (AtCLEs) were calculated (Fig. 2 and Fig. 3). Motifs from the top 5% maximum *d* to AtCLEs were classified into Group “Others”. The rest of the CLE motifs were classified with their closest AtCLE. Consequently, all of the CLE motifs were grouped into six groups, Group1–5 and Others. As a comparison, phylogenetic trees constructed using the *A. thaliana* CLE motifs (Fig. 2a), CLE proteins without signal peptides (Fig. 2b) and log-normalized rank of all-vs-all BLAST e-values of full-length CLE proteins (Fig. 2c) were constructed using the NJ method, as previously described [7, 59]. The new AtCLE clustering using the HCL method was based on the Euclidean distance between each pair of AtCLE motifs (Fig. 2d). The clustering results were similar to the third phylogenetic tree, except for AtCLE8, AtCLE40 and AtCLE43 (Fig. 2 and Additional file 13: Table S2). In Group3, the AtCLE8 and AtCLE12 motifs, which are “RRVPTGPNPLHH” and “RRVPSGPNPLHH”, respectively, share high sequence similarity. However, clustering of AtCLE40 and AtCLE43 was not consistent among the four methods (Fig. 2d). To determine the reasons for these discrepancies, Weblogos of the appropriate subgroups (Group5A and Group5B) were created. Both subgroups were less conserved relative to other subgroups (Additional file 4: Figure S4 and Additional file 14: Table S3).

A cluster tree of all CLE candidates in 69 species was drawn that includes a heatmap indicating the Euclidean distance between the CLE motifs (Fig. 3). The heatmap demonstrated that the CLE candidates in Group5 and Group “Others” have a higher diversity in residual composition. Based on the cluster tree, the 26 AtCLE subgroups were then combined into 11 subgroups. Weblogos of the final 12 subgroups illustrated the importance of “heavy-weight” sites in the classification of CLE motifs (e.g., the 1st and 8th sites (Additional file 4: Figure S4)). Analysis of tandem *CLE* genes revealed that Group1 had the highest rate of tandem genes. Besides, candidates from monocots seemed to form clusters with other monocots, and candidates from dicots seemed to form clusters with other dicots. These data indicate a strong specificity among the monocot CLE motifs and the dicot CLE motifs (Fig. 3). Statistical analysis of the different types of CLE motifs showed that monocots and dicots share very few CLE motifs (18 out of the 474 CLE motifs in dicots). Furthermore, there was no common TDIF/TDIF-like motif shared between monocot and dicot species, probably due to the evolution of distinct vascular patterns in monocots and dicots (Additional file 15: Table S4).

### Evolution of *CLE* genes in plants

**Fig. 4:**
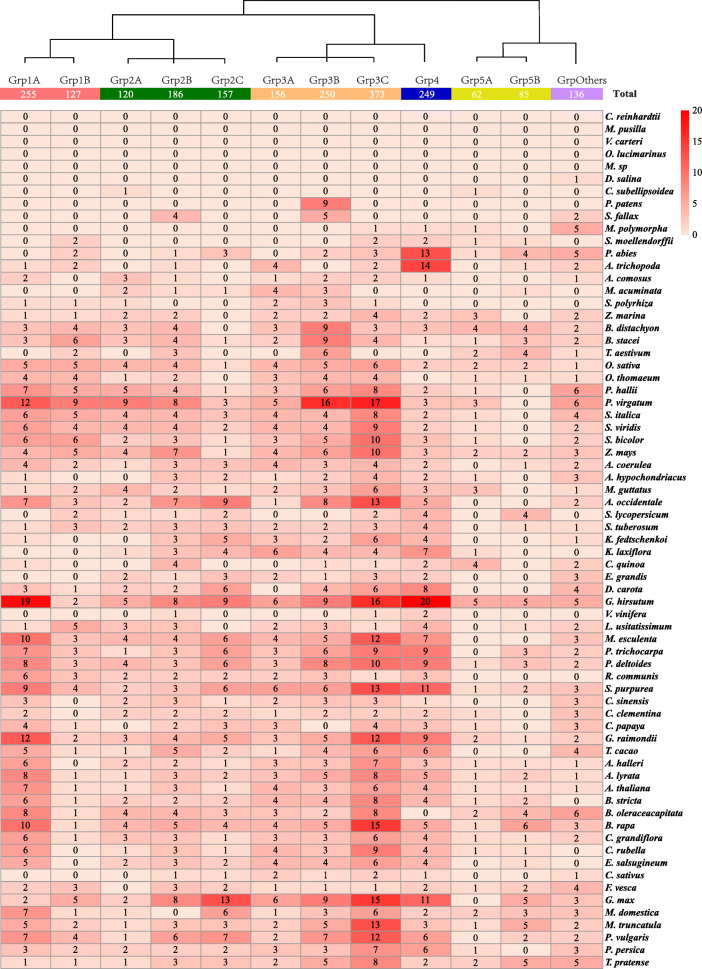
Evolution of *CLE* genes in plants. The number of *CLE* candidate genes from each group in each species was counted and indicated in the grid. The 2156 *CLE* candidates were from 12 groups and 69 species. The abundance of *CLE* candidates in each group is indicated with different shades of red. A darker shade of red indicates more group members. A lighter shade of red indicates fewer group members. The Latin name of each species is indicated on the right. The group name is indicated at the top of the grid. The total number of *CLE* candidates in each subgroup is indicated in the appropriate box. The clustering tree on the top is a simplified version of the tree from Fig. 3

To understand the evolution of *CLE* genes in plants, the number of *CLE* genes in each species was counted (Fig. 4 and Additional file 5: Figure S5). Although three *CLE* genes had been detected in algae, including one in *Dunaliella salina* and two in *Coccomyxa subellipsoidea*, the algal *CLE* genes were atypical because of their low motif scores, low signal peptide scores and poor motif positions (Additional file 16: Table S5). In contrast to algae, there were nine typical *CLE* genes in *Physcomitrella patens* (Fig. 4; Additional file 5: Figure S5 and Additional file 16: Table S5), 11 genes in *Sphagnum fallax* and eight genes in *Marchantia polymorpha* (Fig. 4, Additional file 5: Figure S5). These data provide evidence that *CLE* genes originated in a bryophyte.

The numbers of annotated transcripts in the 62 species of land plants were largely different, ranging from 19,287 (*M. polymorpha*) to 99,386 (*Triticum aestivum*) (Additional file 5: Figure S5). The proportion of *CLE* genes in different species was not fixed, ranging from 0.015% (*Vitis vinifera*) to 0.204% (*Phaseolus vulgaris*). The mean proportion of *CLE* candidates in dicots was slightly higher than in monocots, which were 0.105 and 0.091%, respectively. Their proportions in the three Bryophytes and the pteridophyte (*Selaginella moellendorffii*) were 0.027, 0.041, 0.041 and 0.036%, respectively, in general lower than in the monocots and dicots.

To further investigate the evolution of *CLE* genes in different species, the number of *CLE* genes in each subgroup was counted in each species (Fig. 4). *CLE* candidates appeared in fewer subgroups in lower plants. For example, the nine *CLE* candidates in *P. patens* were all presented in Group3B. *Mapoly1011s0001.1* from *M. polymorpha* was the first candidate identified in Group4. Its motif “HKNPAGPNPIGN” shared high similarity with the CLE motif from Arabidopsis CLE46, a homolog of TDIF. Although none of the Group1 candidates were identified in the bryophytes, two *CLE* candidates from Group1 were identified in *S. moellendorffii*.

In addition, to the finding that CLE motifs are most frequently found in monocots and dicots, the total number of each motif was counted in monocots and/or dicots (Additional file 6: Figure S6 and Additional file 16: Table S5). Our results indicate that the most frequent CLE motif in monocots is “RRVRRGSDPIH”—the same as CLE45 *in A. thaliana*—and that the most frequent CLE motif in dicots is “HEVPSGPNPISN”—the same as CLE41/44 (TDIF) in *A. thaliana*. Monocots and dicots have a strong bias for particular CLE motifs. For example, although the TDIF motif appeared 83 times in dicots, the TDIF motif was not found in monocots. Only a TDIF-like motif “HEVPSGPNPDSN” appeared in monocots (Additional file 17: Table S6).

### Statistics analysis of CLE precursor proteins

**Fig. 5:**
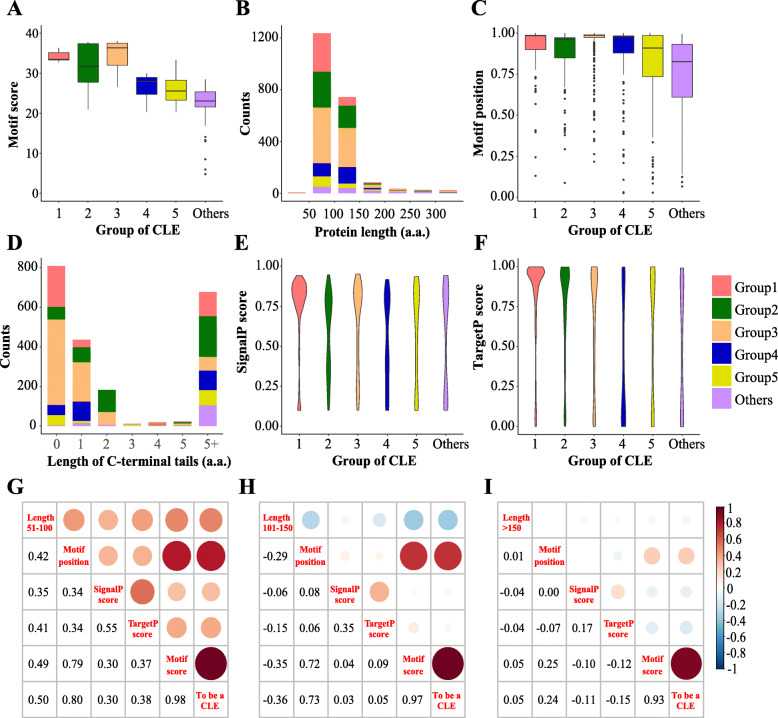
Statistical analysis of the major characteristics of CLE precursors in plants. The major characteristics of 2156 CLE precursor proteins were analyzed, including CLE motif scores (**a**), protein lengths (**b**), CLE motif positions (**c**), lengths of the C-terminal tails (**d**), and SignalP (**e**) and TargetP scores (**f**). Different groups are represented with different colors (**a**-**f**). Histogram: the height of the column represents the CLE candidate counts (**b**, **d**). The line in the box represents the median value. The upper and lower boundary of the box represents the upper and lower quartile values, respectively. The top and bottom of the line represents the maximum and minimum value of non-outliers, respectively. The points represent outliers (**a**, **c**). The widths of the violins represent the distribution density of the indicated value. The tails of the violins were trimmed to match the range of the data (**e**, **f**). **g**-**i** Correlation between the different characteristics of each CLE candidate in three ranges of protein length: 51–100, 101–150 and > 150 amino acid residues, respectively

CLE peptides are derived from nonfunctional precursor proteins by removal of the N-terminal signal peptide from the latter and by enzymatic processing to yield the mature peptide [60]. In order to get a better understanding of CLE protein evolution, various characteristics of different groups were analyzed, including CLE motif score, protein length, relative position of the CLE motif, length of the C-terminal tail, signal peptide scores, and correlations among the major variables of the score matrix (Fig. 5, Additional file 3: Figure S3, Additional file 7: Figure S7 and Additional file 8: Figure S8).

Group 3 had the highest median CLE motif score, followed by the rest of the groups in the following order: Group 1, Group 2, Group 4, Group 5 and Group “Others” (Fig. 5a and Additional file 18: Table S7). Although about 90% of the CLE precursor proteins contain 50–150 amino acid residues, most groups have more candidates containing 50 to 100 residues, except for Group 4 (Fig. 5b and Additional file 9: Figure S9). Group 1 to 4, particularly Group 3, has higher values for the relative position of the CLE motifs (i.e., means closer to the C-terminal end). In contrast, the motif positions in Group 5 and “others” are more widely distributed (Fig. 5c). Approximately two thirds of the candidates have a 0 to 2 residues tail on the C-terminus of the CLE motif. More than 50% of the candidates from both Group 1 and Group 3 did not have a C-terminal tail. The basic amino acids Arginine (R) and lysine (K) dominated at the first amino acid residue position in the short C-terminal tails (1–2 residues), except for the candidates from Group 1 (Fig. 5d and Additional file 7: Figure S7). The presence of a signal peptide in the CLE precursors was predicted online using the SignalP/TargetP server and illustrated with a violin plot. Most genes in Group 1 have high signal peptide scores (Fig. 5e, f). However, about two-thirds of the genes in Grp. 2B and Grp. 5A have SignalP scores lower than the cut-off value (Additional file 19: Table S8). In general, the lengths of the CLE precursor proteins in the bryophytes and *S. moellendorffii* are slightly longer than the average. Other variables, including the signalP score, motif position and the CLE motif score, were not significantly different between vascular and non-vascular plants (Additional file 8: Figure S8).

To determine how much each variable contributed to each CLE candidate, correlations between the five variables and the decision to define a candidate as a CLE were calculated (Fig. 5g-i). Motif score and motif position were decisive factors when the length of the CLE proteins was between 50 and 150 residues. Protein length was positively correlated with the decision when the candidates were shorter than 100 residues. However, the correlation was negative for the candidates between 100 and 150 residues in length (Fig. 5g and h). For longer candidates (> 150 residues), the correlation between motif position and the decision was less. For these candidates, the motif score was the only decisive factor (Fig. 5i). It is worth mentioning that the correlation between the signal peptide scores and the decision was less than expected. In addition, we analyzed the gene structures of the *CLE* candidates in *A. thaliana* and *Zea mays*. The results provide evidence that alternative splicing may allow particular *CLE* genes to concurrently encode proteins with or without the CLE motif (e.g., *CLE46* (AT5G59305) and GRMZM5G875999) (Additional file 10: Figure S10).

### Identification of new types of *CLE* genes

**Fig. 6:**
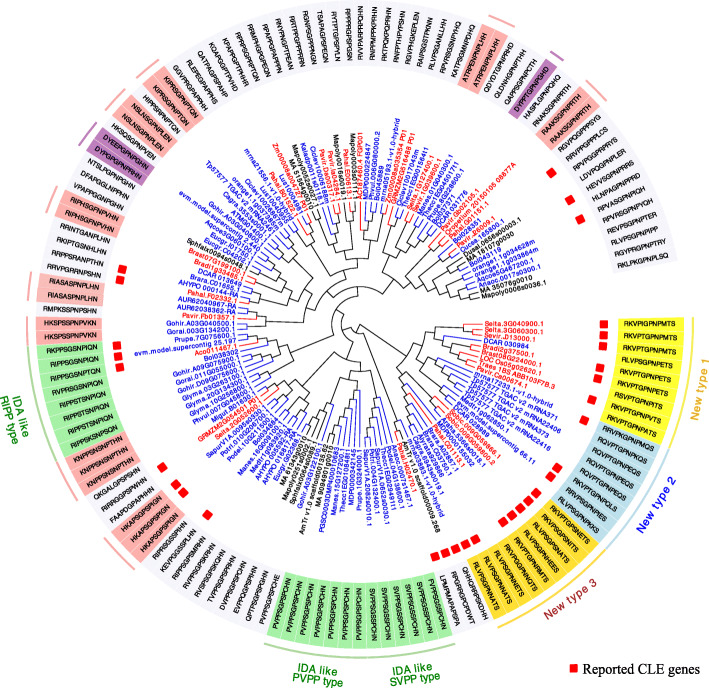
Identification of novel CLE candidates in Group “others”. From the inside to the outside of the ring diagram: clustering tree, gene ID, reporting status, motif sequences, and annotation. The Gene IDs represented in red, blue and black indicate monocot, dicot and other plant species, respectively. Genes that have been reported are marked with red boxes. Candidate motifs of particular interest are highlighted with different colors. New types1, 2 and 3 are highlighted with yellow, light blue and gold, respectively. IDA-like CLE candidates are highlighted with light green. CLE candidates that appeared more than once in Group “others” are labeled with light red. CLE candidates starting with “DY” are indicated with purple

By applying our new approach, 5% (*n* = 136) of the *CLE* candidates that are more distantly related to the Arabidopsis *CLE*s were clustered into Group “others”. A total of 31 of these candidates were reported previously [7]. Based on the clustering, a novel subgroup of candidates (*n* = 26) was identified, with an unusual “serine (S)” at the 12th site of the CLE motif (Fig. 6). This subgroup could be further divided into three types, mainly based on the last three residues of their CLE motifs. All members of this subgroup were from monocots and dicots, consistent with their recent evolution.

**Fig. 7:**
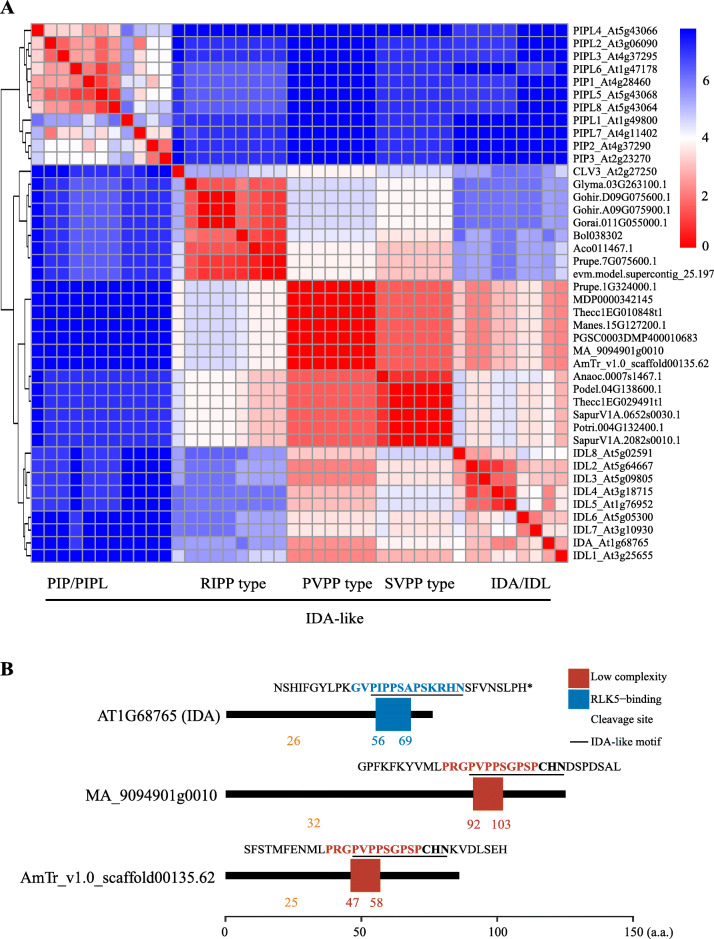
Clustering analysis of IDA-like CLE motifs and Arabidopsis IDA/IDL motifs. **a** Clustering of IDA-like CLE motifs and Arabidopsis IDA/IDL, PIP/PIPL and CLV3 motifs. The heat map indicates the Euclidean distance of each pair of motifs. Red represents short distances. Blue represents long distances. A shorter Euclidean distance implies a higher similarity. **b** Protein domain schematic diagram of *Arabidopsis* IDA and two “PVPP-type” IDA-like CLE candidates. Protein domains were predicted using SMART. Blue box: RLK5-binding domain; red-brown box: low complexity domain; pale-brown triangle: location of the cleavage site of the signal peptide for the secretory pathway; black underline: IDA or IDA-like motif

There were three subsets of *CLE* candidates containing a motif similar to the Arabidopsis secreted peptide IDA “PIPPSAPSKRHN” [19], that we named the SVPP-type (*n* = 6), PVPP-type (*n* = 7) and RIPP-type (*n* = 8), respectively, based on their first four residues (Fig. 6). Most of the IDA/IDL-like candidates were from the monocots and dicots, except for *MA_9094901g0010* and *AmTr_v1.0_scaffold00135.62* from *M. polymorpha* and *Amborella trichopoda*, respectively. By clustering the IDA/IDL-like candidates together with the Arabidopsis IDA/IDL motifs, using PIP/PIPL motifs [61] as the outgroup, we found that the SVPP- and PVPP-type motifs were grouped with the IDA/IDL family, while the RIPP-type motif was more closely related to the CLV3 motif (Fig. 7a). All of the PVPP-type genes were predicted to encode a potential signaling peptide “PVPPSGPSPCHN” (Fig. 7b).

In addition to the novel CLE candidates from Group “others”, small sets of novel candidates were identified in the major groups. The most common residues at the 1st site of a typical CLE motif are arginine (R) and histidine (H). However, candidates with an initial lysine (K) or tryptophan (W) residue in the CLE motif were identified. These K-type and W-type CLE motifs are the most closely related to CLE16 (Group 3C) and CLE45 (Group 2A), respectively (Additional file 11: Figure S11). The 11 K-type candidates were all from monocots. The 13 W-type candidates were exclusively found in dicots.

## Discussions

Small secreted peptides (SSPs) (e.g., CLE peptides) are difficult to predict in silico because their conserved motifs are short, usually less than 20 residues in length. The commonly used method for predicting SSPs use BLAST (Basic Local Alignment Search Tool) [62]. However, when using BLAST, some thresholds should be defined, such as the *S* score, which provides a measure of local similarity for any pair of sequences, and the *E-value*, which is the probability of finding a segment pair with a score no less than the *S* score. It is difficult to define an appropriate threshold for *E-values* when using a CLE as query because it is too short to achieve a high *S* score and therefore, yields a much greater *E-value*. When using a CLE precursor protein as query, the signal peptide and the non-conserved variable region will interfere with the BLAST result. Another common method for predicting SSPs uses HMMER [63]. The latest version is HMMER3 [64]. The results from HMMER depend on the training set. Although the public database of small proteins is expanding, it still cannot meet the demand for predicting SSPs.

In this study, we retrieved all of the annotated amino acid sequences for small proteins from 69 plant species. A CLE-specific score matrix was developed because of the hidden information for peptide processing and peptide–receptor interactions in CLE motifs (Fig. 1). Three ML algorithms were used for predicting *CLE* genes using multiple variables and based on a variety of properties of CLE precursor proteins, in addition to a motif score matrix. A low motif score threshold was set and the union of the ML results was analyzed, in order to keep as many *CLE* candidates as possible. The “low stringency” strategy allowed us to uncover some candidates that are atypical in that they are less similar to the well-studied AtCLEs. By using our newly developed clustering approach for identifying CLE motifs, we were able to classify the major groups and to identify minor groups containing new candidates (Fig. 3, Fig. 4 and Fig. 6). A “high stringency” version of this approach could be developed by simply increasing the threshold of the motif score and changing the ML results from union to intersection.

When a candidate had a low motif score, it probably fell into Group “others” (Fig. 6). Most of the candidates (ca. 78%) in Group “others” have not been previously reported. Several criteria are needed to determine whether a candidate from Group “others” is a CLE, such as including the number of similar motifs, the number of species containing the candidate, and the number of ML algorithms that support the candidate. Candidate motifs that are identified only in one species are more suspicious than candidate motifs that are identified in more than one species.

Although it is possible to classify *CLE* genes based on their contributions to particular biological processes, several difficulties impede a comprehensive functional analysis of *CLE* genes, such as high gene redundancy, specific temporospatial expression patterns and mostly, unknown forms of the mature peptide. Knock-out lines generated using the CRISPR-Cas9 system will shed some light on the biological functions of *CLE* genes. However, this approach is time consuming. Moreover, transgenic manipulation remains difficult in particular species. In contrast, our new clustering method is more efficient because it considers only the amino acid compositions and site weights of CLE motifs. Functional information embedded in the major residues or the heavyweight sites will be reflected in the score matrix and the one-on-one Euclidean distances between the CLE motifs. The clustering results may in turn be helpful for functional analysis of the *CLE* genes that are closely grouped.

Conventional substitution matrices—BLOSUM62, BLOSUM80 and PAM250—are based on massive numbers of amino acid sequences from diverse species. However, using these conventional matrices to identify short amino acid sequences yields results with low statistical confidence, which we have noticed in our efforts to identify *CLE* genes in many different plant species. The ML-assisted method described here was developed specifically for short amino acid sequences, such as SSPs, with high sequence conservativeness and consequently, high residual biasness. Previously existing methods for detecting *CLE* genes did not consider the residual biasness of CLE motifs [7, 58]. Therefore, the flanking sequences of CLE motifs or the full-length amino acid sequences were used to increase the confidence for gene predictions and clustering. In the ML method, other characteristics in addition to the motif sequence of the CLE protein precursors were included. For example, we found that the position of the CLE motif is important for predicting *CLE* genes (Fig. 5g). Based on the comprehensive analysis of various parameters, we conclude that the ML method can give more accurate predictions without too many arbitrary judgments. Consistent with this interpretation, the *CLE* genes predicted with this method have a high degree of consistency with previous reports (Fig. 1e) and also have a good performance in terms of clustering statistics (Additional file 20: Table S9).

A major purpose of this study was to determine how *CLE* genes evolved in plants. We were not able to identify any typical *CLE* genes in the seven species of algae used in this study. The existence of *CLE* genes in *P. patens*, *S. fallax* and *M. polymorpha* provides evidence that the *CLE* genes evolved in bryophytes. All of the nine *P. patens* CLE candidates belong to Group 3B and have a consensus motif sequence of “RXVP(S/T)GPNPLHN”. The motif “RLVPTGPNPLHN” found in *P. patens* is one of the top10 most frequently used CLE motifs in plants, but it is not common in eudicots. A similar motif “RLVPSGPNPLHN”, found in Arabidopsis CLE9/10 was identified exclusively in eudicots. The CLE9/10 motif is the second most abundant CLE motif in dicots. The involvement of *CLE9/10* in the drought response and primary root development in *A. thaliana* [27, 65] is consistent with the peptide “RLVPTGPNPLHN” helping bryophytes to develop adaptations to survive in more arid environments. Another interesting finding in bryophytes is the evolution of the Group 4 candidate *Mapoly1011s0001.1* in *M. polymorpha*. Its potential motif “HKNPAGPNPIGN” is identical to the *Arabidopsis* CLE46 motif “HKHPSGPNPTGN” at all of the conserved sites (Fig. 1b and Additional file 5: Figure S5) [40, 41]. *CLE46* is highly homologous to *CLE41* and *CLE44*, two TDIF encoding genes in *Arabidopsis* [39]. However, similar to other liverworts*, M. polymorpha* has neither vascular tissue nor true roots. Therefore, the presence of a *CLE46*-like gene in *M. polymorpha* remains mysterious. Nevertheless, the number of candidate genes in Group 4 rapidly increased in vascular plants, especially genes encoding candidates with the TDIF motif “HEVPSGPNPISN”. The largest number of candidates in dicots contain the TDIF motif (Additional file 17: Table S6).

Besides the *CLE* gene family, several gene families have been identified that encode SSPs [3, 20]. Among them, the CLEL/GLV/RGF and IDA/IDL motifs share high sequence similarities with the CLE motif [19, 66]. However, our knowledge of the evolutional relationship among these peptide-coding genes remains limited. Based on our less-stringent gene prediction strategy, it is possible to compile a list of atypical *CLE* genes. We found three types of candidates: true *CLE* genes, non-peptide-coding genes and novel peptide-coding genes. We identified 21 candidates that belong to three small but conserved groups in Group “others” (Fig. 6). Their potential CLE motifs are highly similar to the IDA/IDAL motifs and thus, appear to represent a transitional type of CLE and IDA/IDL motif. The *IDA/IDL* genes are involved in floral organ abscission, lateral root emergence and root cap sloughing [67]. Since we have not found any typical *IDA/IDL* genes in *P. patens*, *S. fallax* and *M. polymorpha*, the transitional CLE/IDA candidate *MA_9094901g0010* could be very important for functional and evolutionary studies in the future (Fig. 6 and Fig. 7).

This study was based on a global analysis of the annotated genes from 69 plant species, from single-cell green algae to giant trees. Comparative analysis of *CLE* gene family sequences from multiple species could increase the reliability of gene prediction and characterization and thus, provide information on how these genes have evolved. There are a few challenges remaining for future work. First, the number of lower plant and lower vascular plant species used in this study was limited. The availability of more genome sequences from bryophyte and pteridophyte species will be useful for understanding the origin and evolution of SSP-encoding genes. Second, the quality of genome annotation varies considerably, mainly due to the complexity of each genome and the quality of genome sequencing, assembly and annotation. Thus, high genome complexity or low genomic sequencing quality will increase the frequency of miss counts of SSP-encoding genes. Furthermore, SSP-encoding genes could not be effectively predicted and/or annotated [68]. It is difficult to distinguish them from non-coding sequences because their coding regions are small. More than this, without a reference gene, there is no effective method to predict an SSP-encoding gene when alternative splicing introduces additional complexity. Regarding particular types of SSPs that are variable in length, more research is required to determine how to set a gap penalty for predicting these types of SSPs. The in silico prediction of SSPs that are present in single-copy or low copy numbers (e.g., Casparian Strip Integrity Factor (CIF) from *Arabidopsis*) [69, 70] requires a comparative genomics analysis with multiple species. In addition, integration of next-generation sequencing (NGS)-based transcriptomics and mass spectrometry (MS)-based proteomics analyses will provide essential information about SSPs, especially the novel SSPs.

## Conclusions

In summary, we developed a novel machine learning-aided method for predicting *CLE* genes from 69 plant species by using a CLE motif-specific residual score matrix. We found 2156 CLE candidates, including 627 novel CLE candidates. We also developed a novel clustering method based on the Euclidean distance of CLE motifs in a site-weight dependent manner. Our grouping was relatively consistent with the previous reports by Oelkers et al. [58] and Goad et al. [7] Moreover, the advantage of this new clustering method is that it does not require any flanking sequences from the CLE motifs. Our characterization of CLE candidates provides evidence that approximately 90% of the CLE precursor proteins have a protein length of 50 to 150 amino acid residues, approximately 30% of the CLE candidates may not have a signal peptide targeting them to the secretory pathway, two-thirds of the CLE candidates we identified have a short C-terminal tail (i.e., 0–2 residues) downstream of their CLE motifs, and the CLE motif score was the only decisive factor for identifying candidates longer than 150 residues. These characteristics are important for classifying novel candidates as *CLE* genes. The approach taken here not only helps us to investigate the evolution of the *CLE* gene family, but also allows us to discover a potential evolutionary relationship between the *CLE* and *IDA/IDL* gene families. The IDA/IDL-like CLE candidates represent a missing link between the two families and provide evidence that the *CLE* and *IDA/IDL* genes probably share a common ancestor. Our novel approach for predicting and clustering *CLE* genes may also be applicable to other SSPs and therefore, may provide a powerful tool for studying the origin and evolution of SSPs.

## Methods

### Developing a new residual score matrix for CLE motifs in plants

A new residual score matrix for the CLE motif was developed by integrating the amino acid substitution matrix, the amino acid usage frequency matrix of CLE motifs and the site weights of CLE motifs.

To find an optimal amino acid substitution matrix, three commonly used substitution matrices, BLOSUM62, BLOSUM80 and PAM250, were tested using 116 *CLE* candidates from *A. thaliana*, *O. sativa*, *S. moellendorfii* and *P. abies* (Additional file 20: Table S9). The scores of these 116 reported *CLE* genes followed an order from large to small and were fitted to a curve using the Local Polynomial Regression Fitting (LOESS) method. A matrix with the highest sensitivity was chosen to construct the score matrix for the subsequent analyses.

To develop the amino acid usage frequency matrix for CLE motifs, we used the 1628 reported *CLE* genes as references [7]. The percentage of each amino acid residue *S* at each of the 12 sites of the CLE motif was calculated as follows:

$$ {S}_{ij}=\frac{a_{ij}}{n}\times 100\% $$

where *S*_*ij*_ represents the percentage of amino acid *i* at site *j*; *a*_*ij*_ represents the number of amino acids *i* at site *j* and *n* represents the number of reported *CLE* genes.

The weight of each site (*w*_*j*_) in the CLE motif was based on the *Bits* value of each site [71]. The modified *Bits* values (*Bits’*) were used to assign a weight to each site of the CLE motif with the following steps: (1) select amino acids with *S*_*ij*_ ≥ 25% as the major amino acids for each site, (2) combine *S*_*ij*_ values for these amino acids, and (3) calculate the *Bits’* values based on the ratio of the amino acids at each site using the following equation:

$$ Bits\hbox{'}(j)=\log \kern0.5em {}_2\left(m-k+1\right)-\left({H}_j\hbox{'}+{e}_m\right) $$

$$ {w}_j= Bits\hbox{'}(j)/\max \left( Bits\hbox{'}\right) $$

where *m* represents the types of amino acids (m = 20), *k* represents the number of amino acids with *S*_*ij*_ ≥ 25% at site *j*, *H*_*j*_*’* represents the modified entropy of site *j*, and *e*_*m*_ is the correction number, which was mainly applied when the number of input sequences was less than 20.

A novel residual score matrix *N* was then constructed by integrating the amino acid substitution matrix *M*, the amino acid usage frequency matrix *S* and the site weight *w*_*j*_:

$$ {N}_{ij}={w}_j\times \sum \limits_{k=1}^n\left({S}_{jk}\times {M}_{ik}\right) $$

where *M*_*ik*_ represents the substitution score between amino acid *i* and amino acid *k* in the amino acid substitution matrix, *S*_*jk*_ represents the frequency of amino acid *k* at site *j* in the amino acid usage frequency matrix. The motif score *v* of each CLE motif was calculated by applying the novel score matrix *N*:

$$ v=\sum \limits_{j=1}^{12}{N}_{ij} $$

where *i* represents an amino acid of the CLE motif at site *j.*

### Other variables for the prediction of *CLE* genes

Besides the score of each CLE motif (*v*), other factors were taken into consideration to predict *CLE* genes, including protein length (*L*), signal peptide scores (*SP* for SignalP score/D-value; *TP* for TargetP score/SP-value), and motif position (*P*)*.* Signal peptide scores for each protein sequence was calculated on the SignalP 4.1 Server (http://www.cbs.dtu.dk/services/SignalP/) [72] with a sensitive D-cutoff value (0.34 for SignalP, no TM networks only) and the TargetP 1.1 Server (http://www.cbs.dtu.dk/services/TargetP/) [73] with the plant group. Motif position *P* was calculated as follows:

$$ p=\frac{l_s}{L-11} $$

where *L* represents the length of the corresponding protein and *l*_*s*_ represents the start position of the CLE motif.

### Machine learning aided prediction of *CLE* genes in plants

The coding sequences of 68 species were extracted at the whole genome level from Phytozome v12 (https://phytozome.jgi.doe.gov/pz/portal.html) [74]. Coding sequences of *P. abies* were downloaded from PlantGenIE (http://plantgenie.org) [75, 76]. First, we filtered out protein sequences with *L* < 30 and *L* > 300. For the remaining protein sequences (30 ≤ *L* ≤ 300), we calculated a motif score for any fragment containing 12 amino acid residues. A motif with the highest score was chosen as a potential CLE motif for this protein. The 1529 reported *CLE* genes identified using the HMM algorithms from 53 species in the Phytozome v12.1 database [7] were labeled as *CLE* genes. The number of *CLE* genes (*X*) in a particular species was counted. If *X* ≤ 10, 30 candidates with the highest scores were selected. For a species with *X* > 10, 3*X* candidates with the highest scores were selected. All of the *CLE* genes were removed from the list of candidate genes. The remaining genes were defined as non-*CLE* genes. To build the training data set, the *CLE* and non-*CLE* genes were combined.

Three machine learning algorithms, C4.5, Artificial Neural Network (ANN) and Support Vector Machine (SVM), were used to analyze the training dataset using the above-mentioned five variables. All three algorithms were implemented in the R language (R-3.4.0): C4.5, RWeka-0.4-37 package. The Confidence Pruning Factor *C* was set to 0.01 (ANN, nnet-7.3-12 package). The number of neurons in the hidden layer size was set to 20. The maximum number of iterations was set to 1000 (SVM, e1071_1.6–8 package). The default settings were used for the other parameters. Candidate genes from the remaining 16 species were used for the testing data set. Candidate CLEs were supported by at least one of the three classifiers.

### Clustering of *CLE* genes in plants

The CLE candidates predicted by machine learning were further clustered using a novel protocol based on the Euclidean distance (*d*). The Euclidean distance between each candidate sequence and each reported Arabidopsis CLE motif was calculated to find its minimum distance (*d*_*min*_)*.* The top 5% of motifs with the maximum *d*_*min*_ were categorized into the “others” group. The modified Euclidean distance (*d*) between every two CLE motifs was as follow:

$$ d=\sqrt{\sum \limits_{j=1}^{12}{d_j}^2} $$

$$ {d}_j=\left\{\begin{array}{l}0\kern2.1em \left({a}_j={b}_j\right)\\ {}{w}_j\kern1em \left({a}_j\ne {b}_j\right)\end{array}\right. $$

Where *a*_*j*_ represents the amino acid at site *j* of a candidate CLE motif and *b*_*j*_ represents the amino acid at site *j* of *A. thaliana* CLE motifs. The distance between *a*_*j*_ and *b*_*j*_ was defined as *d*_*j*_.

For all grouped *CLE* candidate genes, a hierarchical clustering (HCL) method was applied with R (R-3.4.0) to build a clustering tree. Phylogenetic trees of *A. thaliana* CLE motifs, full-length CLE proteins without signal peptides and log-normalized rank of all-vs-all BLAST e-values were constructed using the neighbor-joining (NJ) method with MEGA X [77]. The clustering trees and phylogenetic trees were edited using Evolview (http://www.evolgenius.info/evolview/) [78].

### Statistical analysis

To find out the bias in amino acid usage in CLE precursor proteins and CLE motifs, the amino acid composition of all proteins, all small proteins (i.e., proteins with lengths between 50 and 200 amino acid residues) and all CLE candidates were analyzed in 69 plant species. To study the evolution of the *CLE* genes, the numbers of *CLE* candidate genes were counted in each species and in each group. CLE precursor proteins were characterized by analyzing the distribution of motif scores, protein lengths, motif positions, lengths of C-terminal tails, SignalP and TargetP scores of each CLE candidate by group. Decisive factors in identifying a *CLE* candidate gene were uncovered using a correlation analysis between each of the above-mentioned variables and the decision in three ranges of protein lengths, 51–100, 101–150 and > 150 amino acid residues. The clustering trees of CLE candidates in Group “others” and IDA-like candidates were built by applying the HCL method—based on Euclidean distance—to every pair of candidate sequences. The lengths of the C-terminal tails and their corresponding amino acid compositions in each subgroup were evaluated using a heatmap that showed the counts of CLE candidates with different lengths of C-terminal tails. Weblogos were used to represent the conserved residues. For *A. thaliana* and *Z. mays*, the gene structures of the *CLE* candidates with alternative splicing were obtained from the gff3 files of *A. thaliana* and *Zea mays* in Phytozome v12 (https://phytozome.jgi.doe.gov/pz/portal.html).

Weblogo (http://weblogo.threeplusone.com/) [79] was used to create the sequence logo. MEME (http://meme-suite.org/tools/meme) [80] was used to calculate the e-values of each CLE motif. The R package pheatmap-1.0.17, corrplot-0.84 and UpSetR-1.4.0 were applied to create heat maps, correlation maps and upset plots, respectively. Other plots were created using ggplot2–3.2.0. All data were processed using the R language (R-3.4.0 and R-3.6.1).

## Supplementary information

**Additional file 1: Figure S1.** Amino acid composition of all proteins, all small proteins, CLE precursors, CLE signal peptides, CLE variable regions and CLE motifs in 69 species.

**Additional file 2: Figure S2.** Comparison of three amino acid substitution matrices for evaluating CLE motif scores.

**Additional file 3: Figure S3.** Amino acid usage frequency of CLE motifs.

**Additional file 4: Figure S4.** Weblogo representation of CLE motifs in each group or subgroup of the *CLE* gene family in plants.

**Additional file 5: Figure S5.** Number and proportion of *CLE* genes in the genomes of 69 plant species.

**Additional file 6: Figure S6.** Top ten most frequently used CLE motifs in 69 species.

**Additional file 7: Figure S7.** Statistical analysis of the lengths of the C-terminal tails of CLE candidates.

**Additional file 8: Figure S8.** Statistical analysis of protein lengths, SignalP scores, motif positions and CLE motif scores of CLE candidates from 69 species.

**Additional file 9: Figure S9.** Distribution of protein lengths of CLE precursors in the range of 50–150 amino acid residues.

**Additional file 10: Figure S10.** Gene structure of *CLE* candidates regulated by alternative splicing in *A. thaliana* and *Z. mays*.

**Additional file 11: Figure S11.** K-type and W-type CLE candidates in plants.

**Additional file 12: Table S1.** Predicted CLE candidates in 69 plant species

**Additional file 13: Table S2.** Comparison of group information on Arabidopsis CLE genes from the literature and from this study.

**Additional file 14: Table S3.** Comparison of E-values for evaluating the grouping of CLE motifs from Goad et al. [7] and from the site-weight based method described in this study.

**Additional file 15: Table S4.** Number of types of CLE motifs in 69 plant species.

**Additional file 16: Table S5.** CLE candidates in algae and bryophytes.

**Additional file 17: Table S6.** Top 10 most frequently used CLE motifs in monocots and dicots.

**Additional file 18: Table S7.** Number of CLE genes lying above the CLE motif scores of the corresponding quantiles.

**Additional file 19: Table S8.** Number of CLE genes lying above the threshold of the SignalP score.

**Additional file 20: Table S9.** CLE motifs used for finding the optimal amino acid substitution matrix.

## Data Availability

All data generated or analysed during this study are included in this published article and its supplementary information files.
