# Correction to: Predicting and clustering plant *CLE* genes with a new method developed specifically for short amino acid sequences

**DOI:** 10.1186/s12864-020-07231-4

**Published:** 2020-12-18

**Authors:** Zhe Zhang, Lei Liu, Melis Kucukoglu, Dongdong Tian, Robert M. Larkin, Xueping Shi, Bo Zheng

**Affiliations:** 1 Key Laboratory of Horticultural Plant Biology of Ministry of Education, Huazhong Agricultural University, Wuhan, 430070 China; 2 College of Horticulture and Forestry Sciences, Huazhong Agricultural University, Wuhan, 430070 China; 3 Institute of Biotechnology, Helsinki Institute of Life Science (HILIFE), University of Helsinki, 00014 Helsinki, Finland; 4 Viikki Plant Science Centre, University of Helsinki, 00014 Helsinki, Finland

**Correction to: BMC Genomics 21, 709 (2020)**

**https://doi.org/10.1186/s12864-020-07114-8**

Following the publication of the original article [1], it was reported that there was an error in Fig. 7 whereby the yellow triangles showing the cleavage sites were not present. The corrected Fig. 7 is included in this Correction article, and the original article has been corrected.

**Fig. 7:**
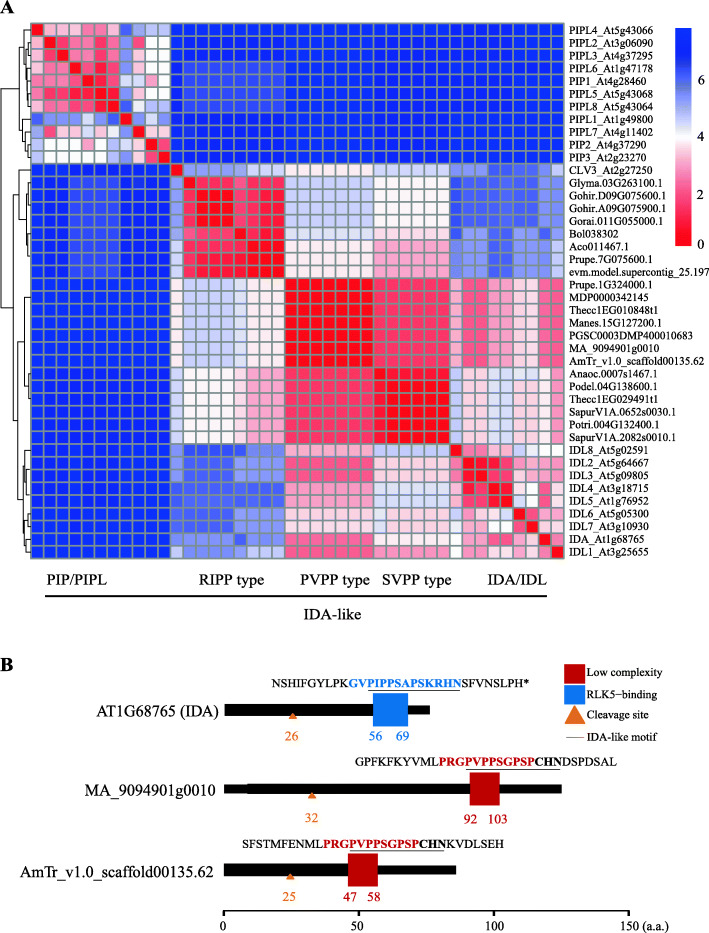
Clustering analysis of IDA-like CLE motifs and Arabidopsis IDA/IDL motifs. **a** Clustering of IDA-like CLE motifs and Arabidopsis IDA/IDL, PIP/PIPL and CLV3 motifs. The heat map indicates the Euclidean distance of each pair of motifs. Red represents short distances. Blue represents long distances. A shorter Euclidean distance implies a higher similarity. **b** Protein domain schematic diagram of *Arabidopsis* IDA and two “PVPP-type” IDA-like CLE candidates. Protein domains were predicted using SMART. Blue box: RLK5-binding domain; red-brown box: low complexity domain; pale-brown triangle: location of the cleavage site of the signal peptide for the secretory pathway; black underline: IDA or IDA-like motif
