# Supplementary material for: Predicting and clustering plant CLE genes with a new method developed specifically for short amino acid sequences
Source: BMC Genomics. 2020 Oct 12;21:709. doi: 10.1186/s12864-020-07114-8 (PMC7552357; doi:10.1186/s12864-020-07114-8)
Supplement: Supplementary file 1 — Additional file 1: Figure S1. Amino acid composition of all proteins, all small proteins, CLE precursors, CLE signal peptides, CLE variable regions and CLE motifs in 69 species. [file 12864_2020_7114_MOESM1_ESM.pdf]

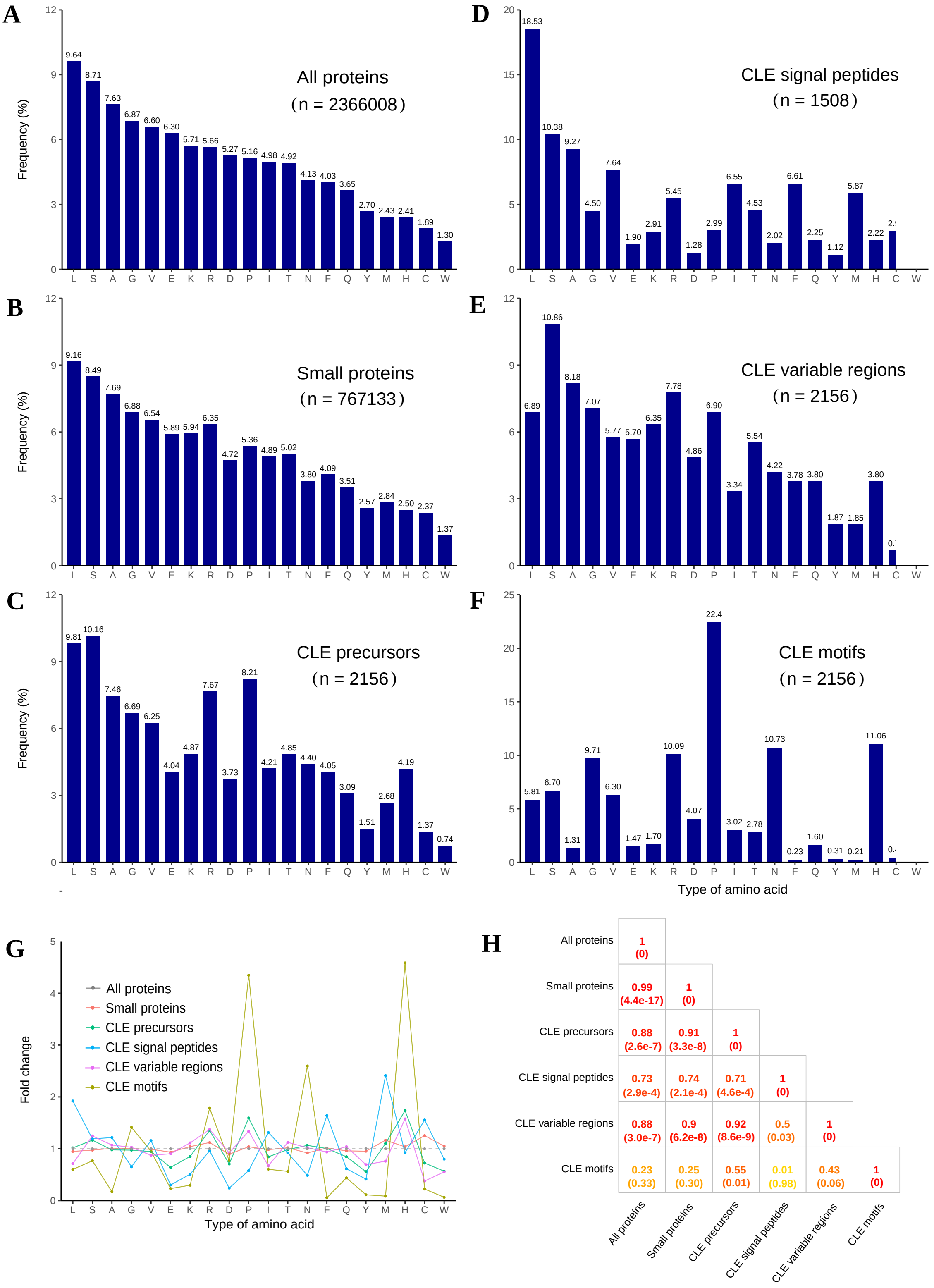

Figure S1 Amino acid composition of all proteins, all small proteins, CLE precursors, CLE signal peptides, CLE variable regions and CLE motifs in 69 species

Amino acid composition statistics of all proteins (**A**), all small proteins (**B**), CLE precursors (**C**), CLE signal peptides (**D**), CLE variable regions (**E**), CLE motifs (**F**) and fold changes of each type of amino acid in comparison with the amino acid composition of all proteins (**G**). (**H**) Pearson correlation coefficient and *p-value* for correlation test (in brackets) of amino acid ratio of all proteins, all small proteins, CLE precursors, CLE signal peptides, CLE variable regions and CLE motifs.
