# Supplementary material for: Predicting and clustering plant CLE genes with a new method developed specifically for short amino acid sequences
Source: BMC Genomics. 2020 Oct 12;21:709. doi: 10.1186/s12864-020-07114-8 (PMC7552357; doi:10.1186/s12864-020-07114-8)
Supplement: Supplementary file 2 — Additional file 2: Figure S2. Comparison of three amino acid substitution matrices for evaluating CLE motif scores. [file 12864_2020_7114_MOESM2_ESM.pdf]

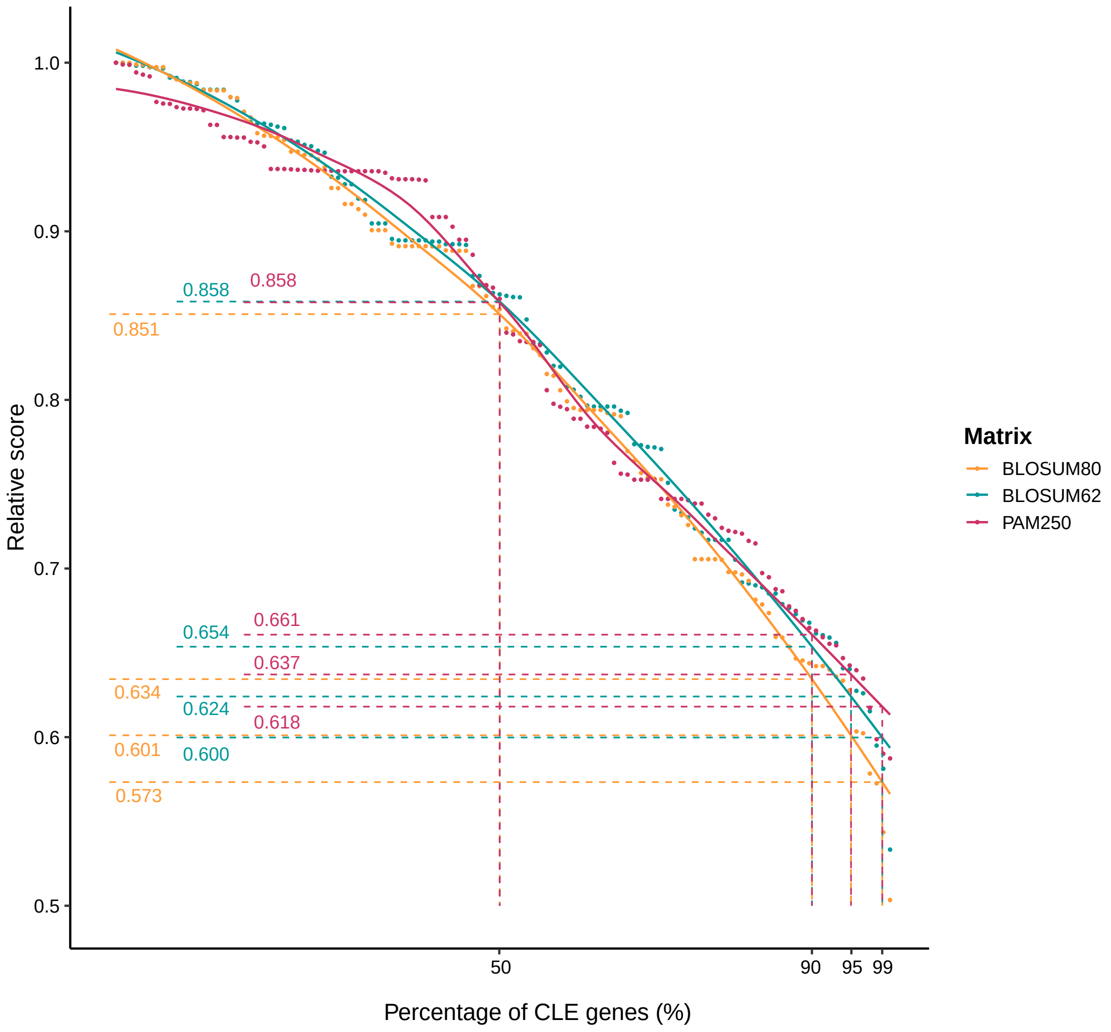


**Figure S2 Comparison of three amino acid substitution matrices for evaluating CLE motif scores**

The dots revealed the relative score (CLE motif score/max CLE motif score) of each CLE motif in *A. thaliana*, *O. sativa*, *S. moellendorfii* and *P. abies*. Three different amino acid substitution matrices were tested, including BLOSUM80, BLOSUM62 and PAM250. All dots (relative scores) were ranked from large to small, and fitted into a curve using Local Polynomial Regression Fitting (LOESS) method. The numbers reflect the relative score at the range of 50, 90, 95 and 99% of all tested motifs, respectively. Different colors represent different amino acid substitution matrices: yellow, BLOSUM80; green, BLOSUM62; red, PAM250.
