# Supplementary material for: Predicting and clustering plant CLE genes with a new method developed specifically for short amino acid sequences
Source: BMC Genomics. 2020 Oct 12;21:709. doi: 10.1186/s12864-020-07114-8 (PMC7552357; doi:10.1186/s12864-020-07114-8)
Supplement: Supplementary file 3 — Additional file 3: Figure S3. Amino acid usage frequency of CLE motifs. [file 12864_2020_7114_MOESM3_ESM.pdf]

Type of amino acid \ Site of CLE motif	1	2	3	4	5	6	7	8	9	10	11	12
**A**	0.13	2.58	2.58	0.19	2.32	1.42	3.10	0.39	1.68	0.39	0.39	0.26
**R**	85.99	21.37	0.26	6.20	6.33	0.19	0.19	0.19	1.16	2.71	0.32	0.00
**N**	0.00	0.26	0.06	0.00	8.78	0.00	0.00	50.03	0.06	0.13	0.06	66.49
**D**	0.26	0.71	0.00	0.00	0.19	0.00	0.00	47.06	0.13	1.61	0.13	0.19
**C**	0.06	0.00	0.00	0.00	0.19	3.94	0.00	0.00	0.00	0.00	0.06	0.00
**Q**	0.26	2.13	0.06	0.13	4.20	0.00	0.00	0.00	0.32	12.20	0.65	0.00
**E**	0.06	13.11	0.00	0.00	0.52	0.19	0.00	0.06	0.00	2.26	0.26	0.06
**G**	0.19	1.48	0.19	0.06	21.43	90.57	0.13	0.19	0.39	0.13	1.48	0.19
**H**	9.68	0.13	0.06	3.81	0.00	0.00	0.00	0.00	0.71	2.39	87.15	31.38
**I**	0.06	3.49	8.20	0.52	0.00	0.00	0.00	0.00	0.13	24.47	0.00	0.13
**L**	0.32	24.53	0.00	0.06	0.06	0.13	0.26	0.00	0.45	49.19	0.06	0.19
**K**	0.97	11.17	0.06	0.84	2.32	0.00	0.06	0.00	0.39	2.00	0.00	0.19
**M**	0.19	1.74	0.00	0.06	0.19	0.06	0.00	0.00	0.00	0.06	0.06	0.00
**F**	0.00	1.81	0.13	0.45	0.13	0.06	0.13	0.00	0.00	0.26	0.06	0.06
**P**	0.26	1.10	0.71	84.64	0.32	0.19	79.79	0.45	94.00	0.19	0.26	0.13
**S**	0.13	2.78	17.82	0.52	27.31	2.84	15.69	1.10	0.26	0.71	8.72	0.39
**T**	0.26	3.62	0.84	0.00	25.37	0.13	0.39	0.13	0.13	0.32	0.19	0.13
**W**	1.10	0.00	0.00	0.00	0.00	0.06	0.00	0.06	0.00	0.06	0.00	0.00
**Y**	0.00	0.06	0.06	2.39	0.13	0.00	0.26	0.26	0.00	0.06	0.06	0.00
**V**	0.06	7.81	68.95	0.13	0.19	0.19	0.00	0.06	0.19	0.84	0.06	0.13

Figure S3 Amino acid usage frequency of CLE motifs

Amino acid one letter codes were listed on the left. Each number in the grid represents the frequency of a certain amino acid at the corresponding site.
