# Supplementary material for: Predicting and clustering plant CLE genes with a new method developed specifically for short amino acid sequences
Source: BMC Genomics. 2020 Oct 12;21:709. doi: 10.1186/s12864-020-07114-8 (PMC7552357; doi:10.1186/s12864-020-07114-8)
Supplement: Supplementary file 4 — Additional file 4: Figure S4. Weblogo representation of CLE motifs in each group or subgroup of the CLE gene family in plants. [file 12864_2020_7114_MOESM4_ESM.pdf]

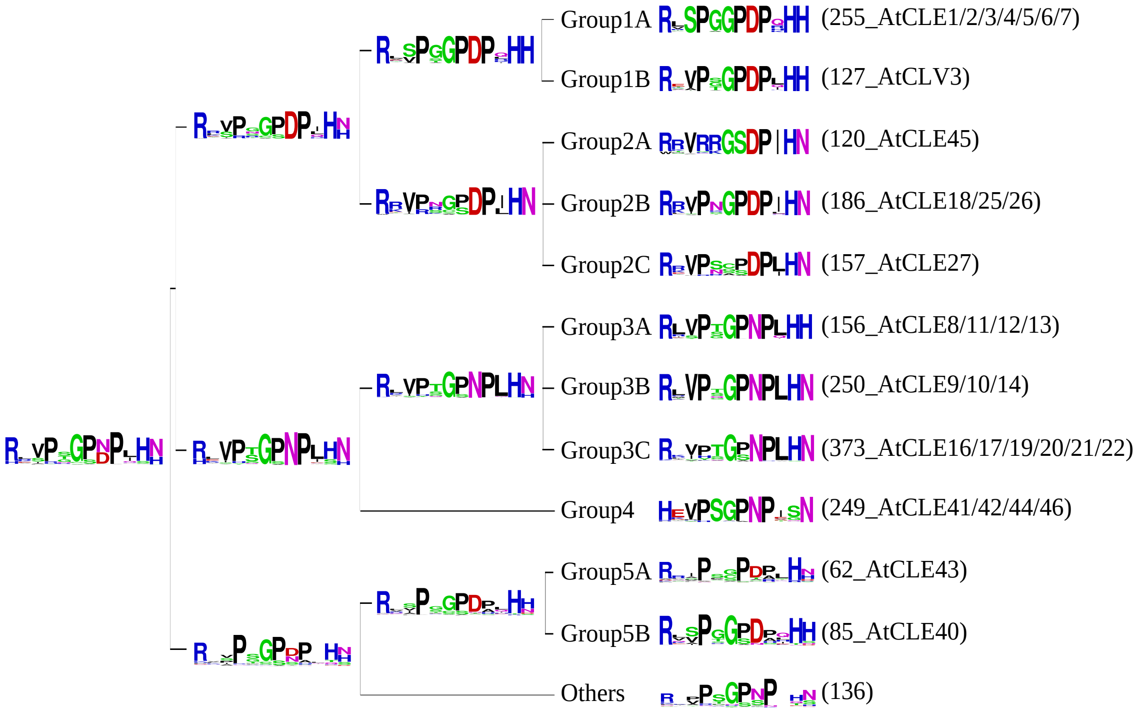

Figure S4 Weblogo representation of CLE motifs in each group or subgroup of the *CLE* gene family in plants

Weblogos were created for each major node of the cluster tree (see Figure 3). For each of the twelve groups, the total number of CLE motifs and the name of corresponding *Arabidopsis* CLEs were shown in the parentheses on the right.
