# Supplementary material for: Predicting and clustering plant CLE genes with a new method developed specifically for short amino acid sequences
Source: BMC Genomics. 2020 Oct 12;21:709. doi: 10.1186/s12864-020-07114-8 (PMC7552357; doi:10.1186/s12864-020-07114-8)
Supplement: Supplementary file 5 — Additional file 5: Figure S5. Number and proportion of CLE genes in the genomes of 69 plant species. [file 12864_2020_7114_MOESM5_ESM.pdf]

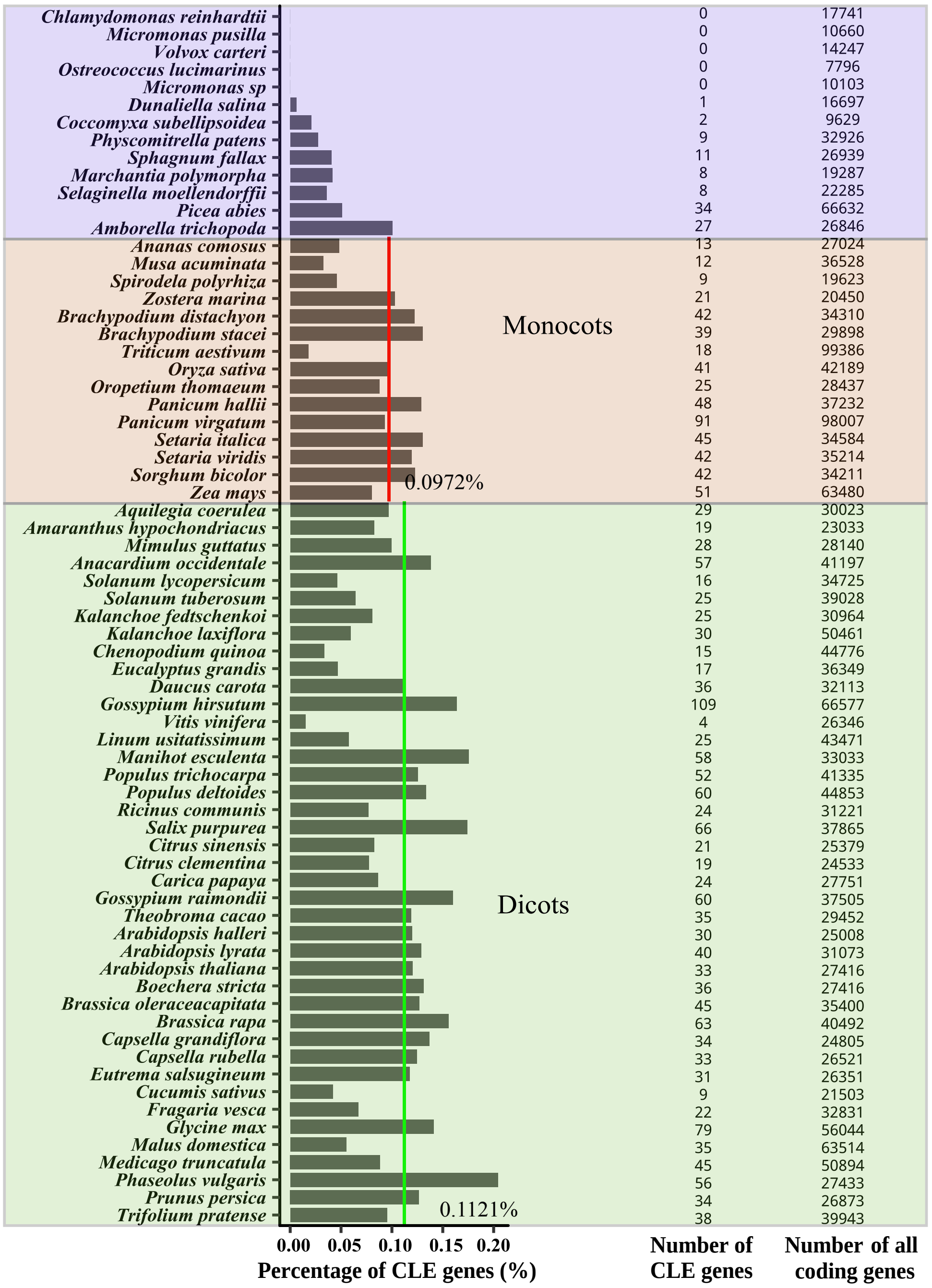

Figure S5 The number and the proportion of *CLE* genes in the genomes of 69 plant species

From the left to the right: Latin names of plant species, the percentage of CLE genes in the genome of each species, the total number of CLE genes in the genome of each species, and the total number of genes in the genome of each species.The background colors represent the taxonomy of species: light green, dicots; sand color, monocots; purple, other species. The two vertical lines in the histogram represent the median value of the percentage of *CLE* genes in the genome, red for the monocots and green for the dicots.
