# Supplementary material for: Predicting and clustering plant CLE genes with a new method developed specifically for short amino acid sequences
Source: BMC Genomics. 2020 Oct 12;21:709. doi: 10.1186/s12864-020-07114-8 (PMC7552357; doi:10.1186/s12864-020-07114-8)
Supplement: Supplementary file 6 — Additional file 6: Figure S6. Top ten most frequently used CLE motifs in 69 species. [file 12864_2020_7114_MOESM6_ESM.pdf]

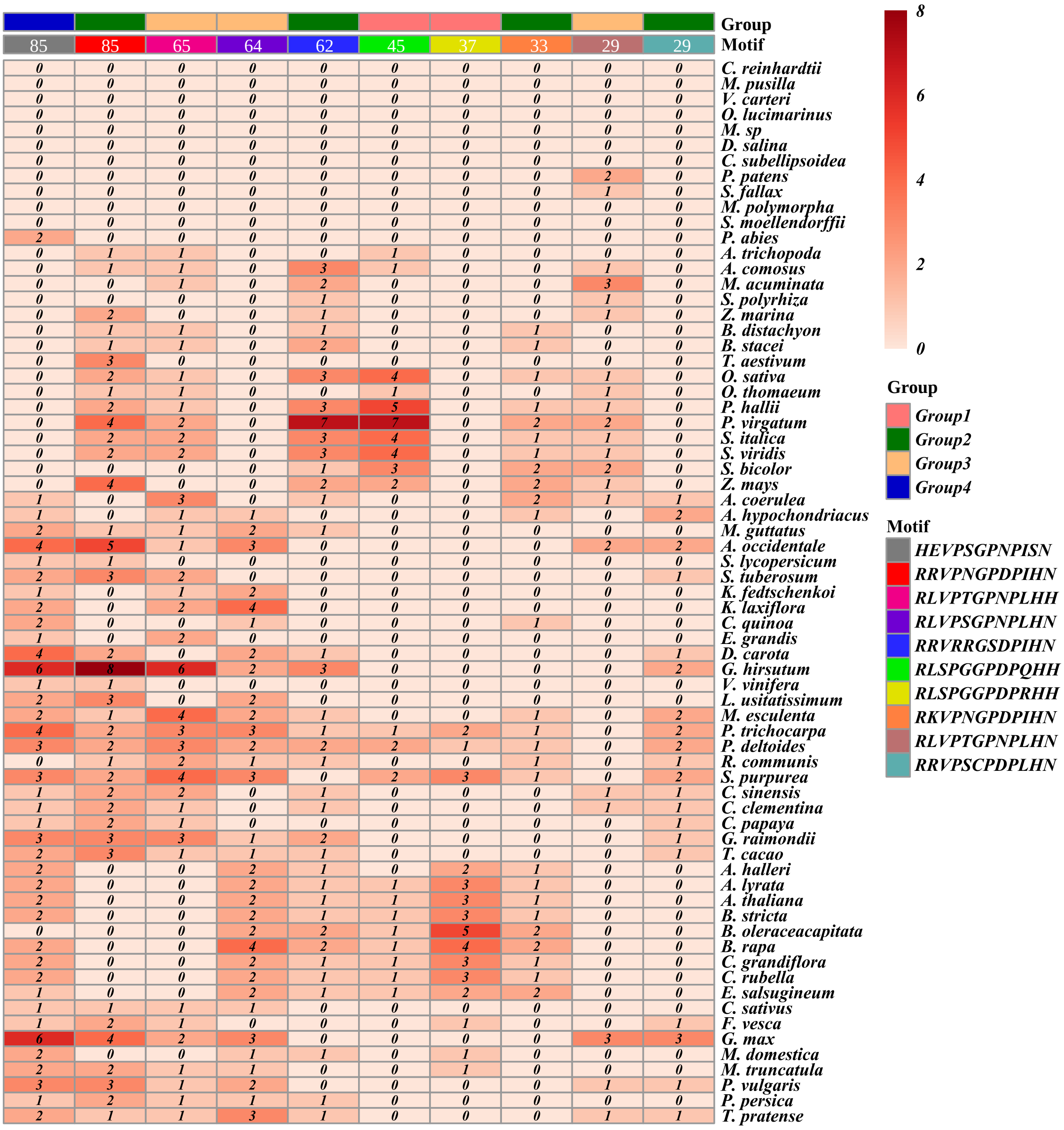

Figure S6 The top ten most frequently used CLE motifs in 69 species

Ten most frequently used CLE motifs were selected based on their total counts in the genomes of 69 plant species. A heatmap in 10 x 69 grids was created to display the counts of each motif in each species. The counts of CLE candidates were indicated by using the gradient in red: dark red means more and light red means less. The Latin name of each species and the motif sequences were listed on the left; the total counts of each CLE motif were shown on the top.
