# Supplementary material for: Predicting and clustering plant CLE genes with a new method developed specifically for short amino acid sequences
Source: BMC Genomics. 2020 Oct 12;21:709. doi: 10.1186/s12864-020-07114-8 (PMC7552357; doi:10.1186/s12864-020-07114-8)
Supplement: Supplementary file 7 — Additional file 7: Figure S7. Statistical analysis of the lengths of the C-terminal tails of CLE candidates. [file 12864_2020_7114_MOESM7_ESM.pdf]

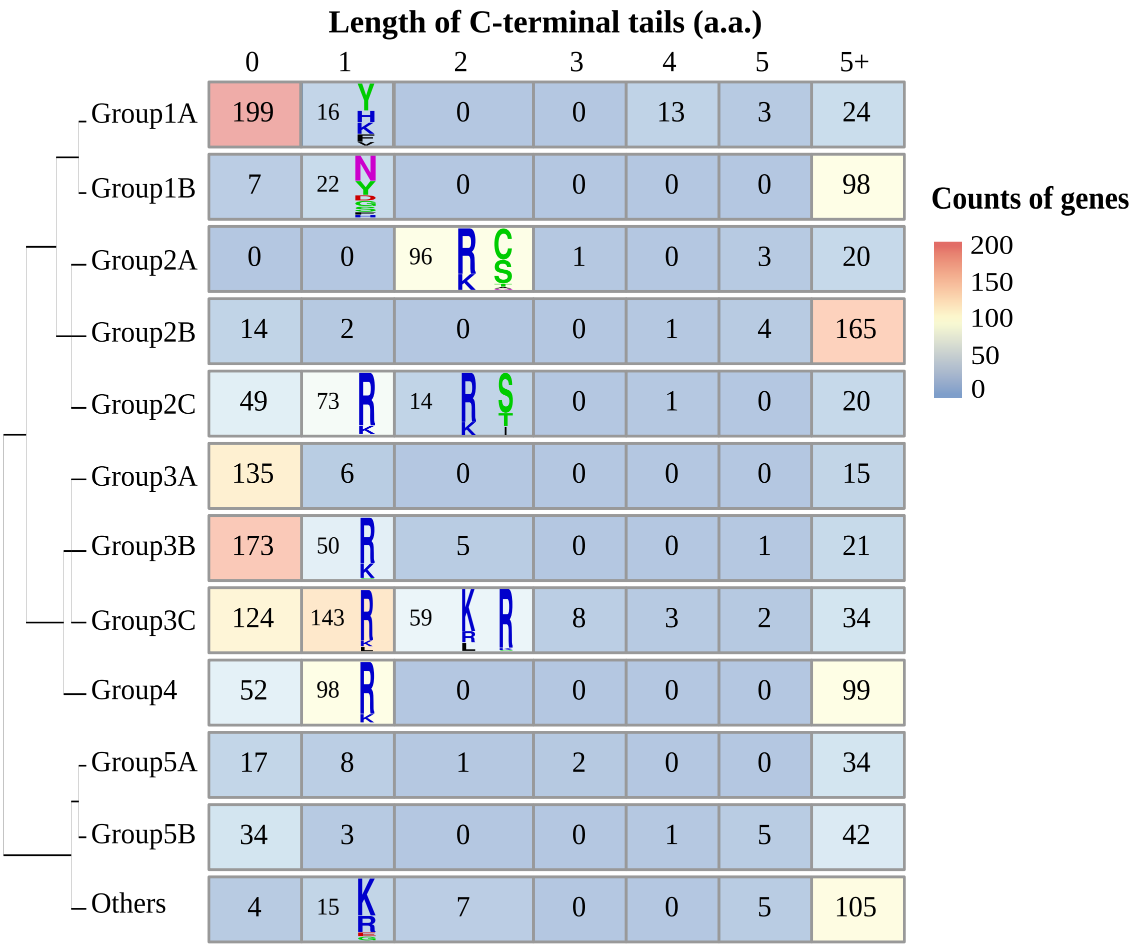

Figure S7 Statistical analysis of the lengths of the C-terminal tails of CLE candidates

A heatmap in 7 x 12 grids was created to display the number of CLE candidates in different lengths of the C-terminal tails (abscissa) and in different subgroups of CLE family (ordinate). Length of the C-terminal tails = 0 a.a. means no C-terminal tail after the CLE motif. Under the circumstance of tail = 1 or 2 a.a., Weblogo representation of the tails were created to show the corresponding amino acid composition if the number of CLE candidates was more than 10.
