# Supplementary material for: Predicting and clustering plant CLE genes with a new method developed specifically for short amino acid sequences
Source: BMC Genomics. 2020 Oct 12;21:709. doi: 10.1186/s12864-020-07114-8 (PMC7552357; doi:10.1186/s12864-020-07114-8)
Supplement: Supplementary file 8 — Additional file 8: Figure S8. Statistical analysis of protein lengths, SignalP scores, motif positions and CLE motif scores of CLE candidates from 69 species. [file 12864_2020_7114_MOESM8_ESM.pdf]

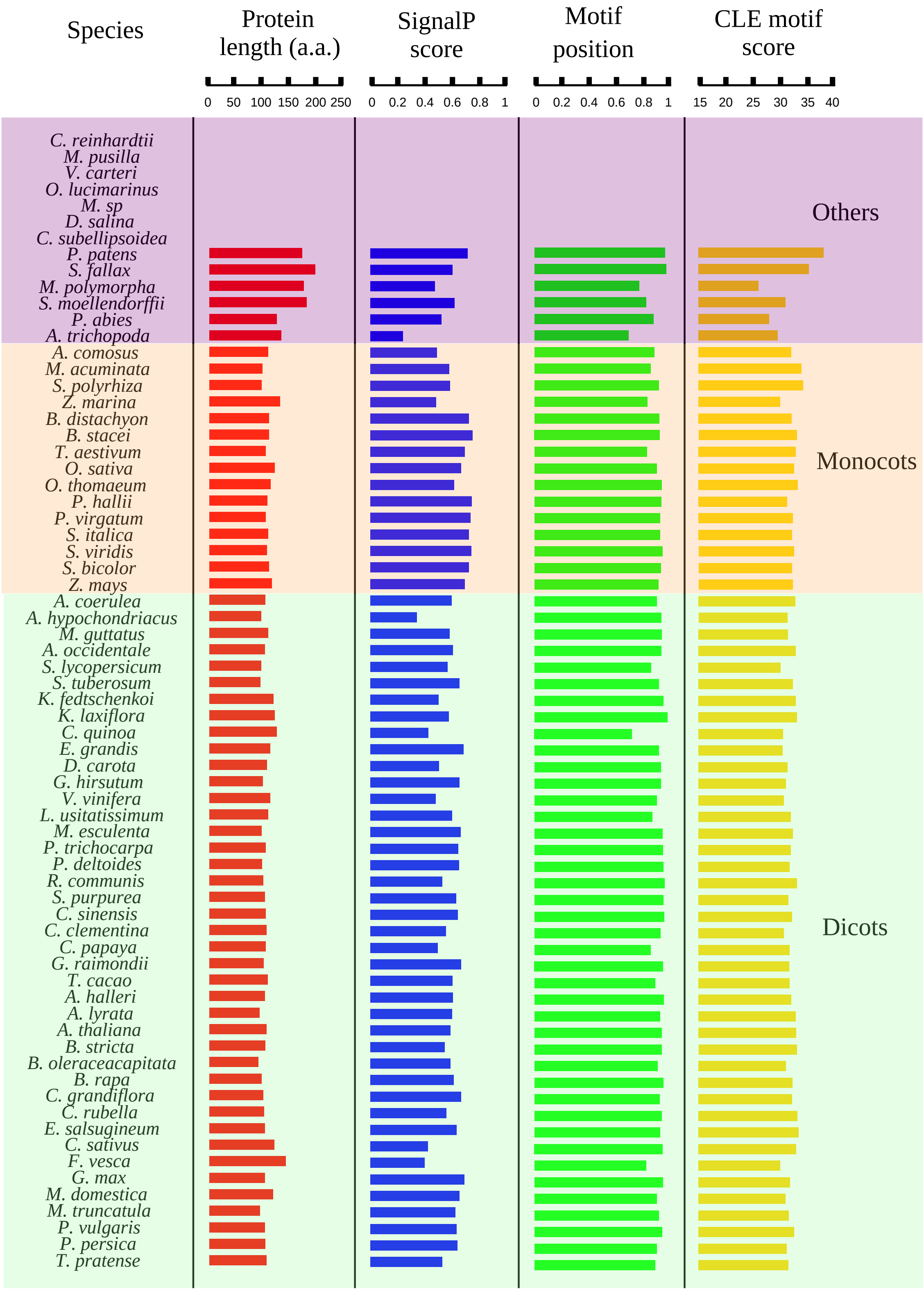

Figure S8 Statistical analysis of protein length, SignalP score, motif position and CLE motif score of CLE candidates in 69 species

From the left to the right, the histograms showed the median of protein length, mean of SignalP score, mean of motif position and mean of CLE motif score of CLE candidates in 69 species. Background colors represent the taxonomy of species: light green, dicots; light yellow, monocots; purple, other species.
