# Supplementary figures and images for: Predicting and clustering plant CLE genes with a new method developed specifically for short amino acid sequences
Source: BMC Genomics. 2020 Oct 12;21:709. doi: 10.1186/s12864-020-07114-8 (PMC7552357; doi:10.1186/s12864-020-07114-8)

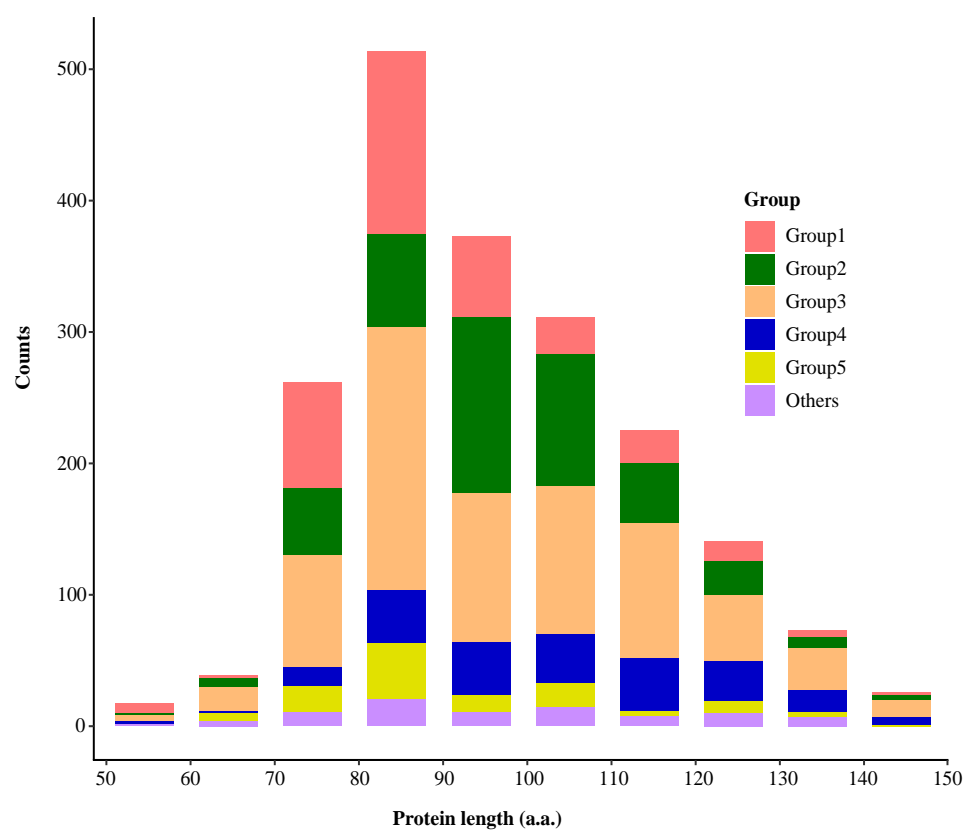

**Figure S9** Distribution of protein lengths of CLE precursors in the range of 50-150 a.a

Supplement: Supplementary file 9 — Additional file 9: Figure S9. Distribution of protein lengths of CLE precursors in the range of 50–150 amino acid residues. [file 12864_2020_7114_MOESM9_ESM.pdf]
