# Supplementary material for: Predicting and clustering plant CLE genes with a new method developed specifically for short amino acid sequences
Source: BMC Genomics. 2020 Oct 12;21:709. doi: 10.1186/s12864-020-07114-8 (PMC7552357; doi:10.1186/s12864-020-07114-8)
Supplement: Supplementary file 10 — Additional file 10: Figure S10. Gene structure of CLE candidates regulated by alternative splicing in A. thaliana and Z. mays. [file 12864_2020_7114_MOESM10_ESM.pdf]

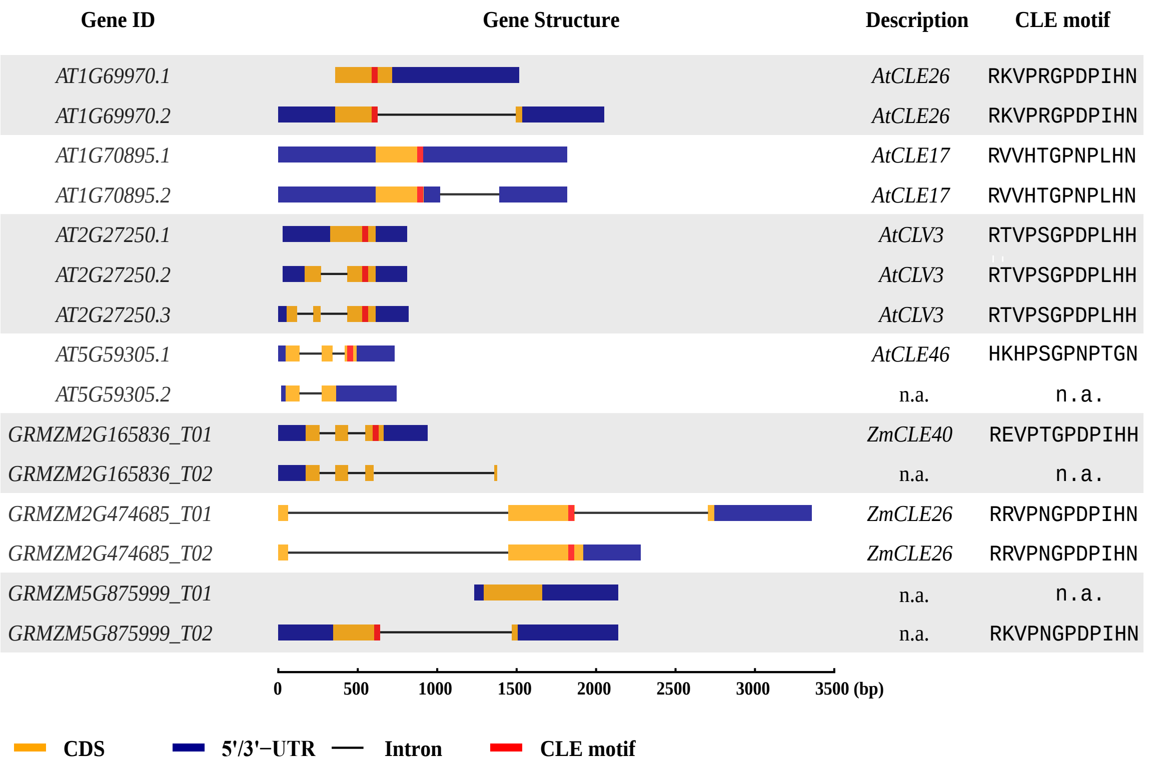

Figure S10 Gene structure of *CLE* candidates containing alternative splicing in *A. thaliana* and *Z. may*

From the left to the right: gene ID, gene structure, gene description and motif sequences of CLE candidates. containing alternative splicing in *A. thaliana* and *Z. may*. Gene structure was depicted using ggplot2-3.2.0. Orange box, CDs; navy blue box, UTR; red box, CLE motif; black line, intron; n.a., information not available.
