# Supplementary material for: Predicting and clustering plant CLE genes with a new method developed specifically for short amino acid sequences
Source: BMC Genomics. 2020 Oct 12;21:709. doi: 10.1186/s12864-020-07114-8 (PMC7552357; doi:10.1186/s12864-020-07114-8)
Supplement: Supplementary file 11 — Additional file 11: Figure S11. K-type and W-type CLE candidates in plants. [file 12864_2020_7114_MOESM11_ESM.pdf]

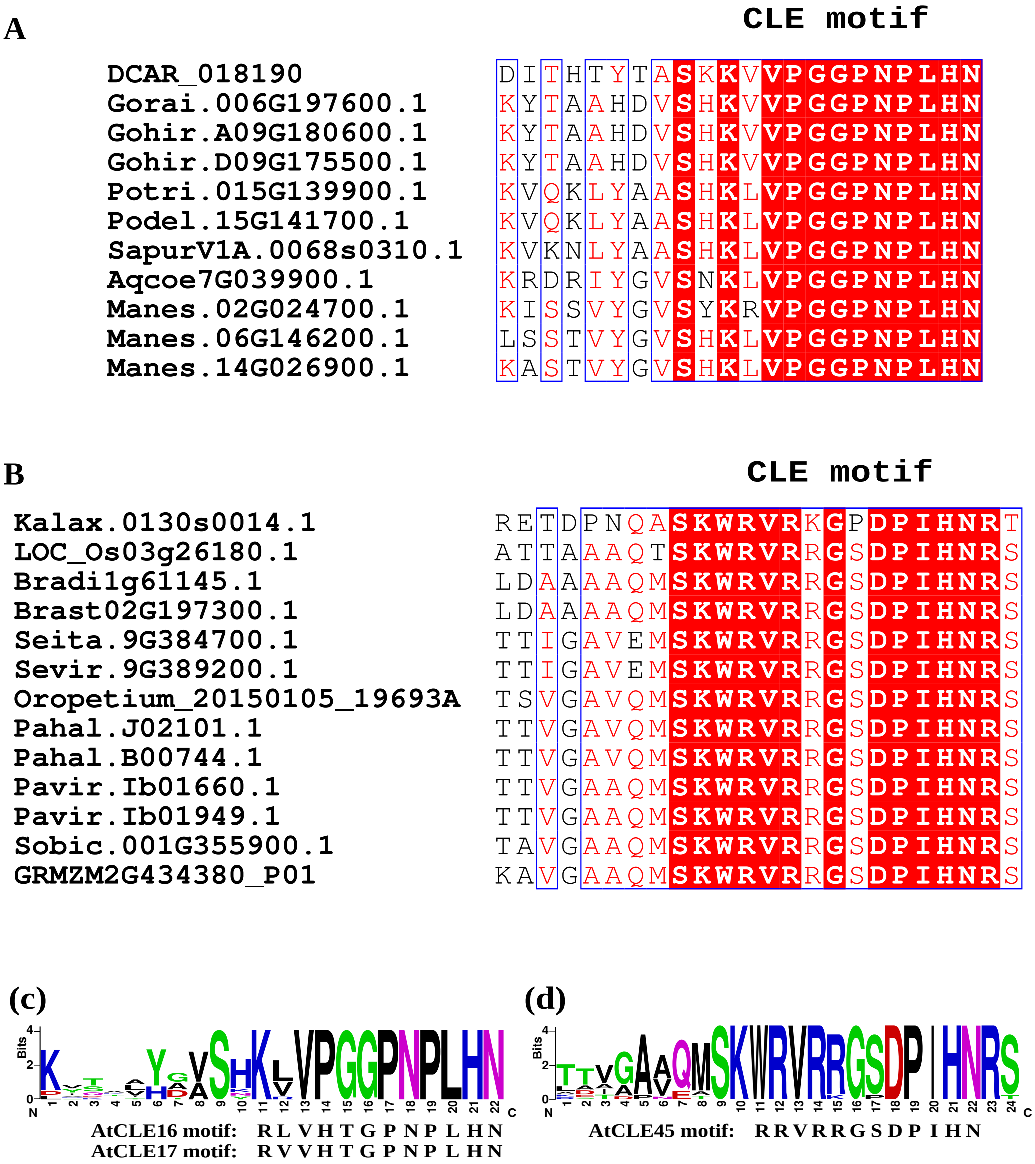

Figure S11 K-type and W-type CLE candidates in plants

(**A, B**) Alignment of K-type and W-type CLE candidates, respectively. CLE motifs were indicated by blue lines. (**C, D**) Weblogo representation of K-type and W-type CLE candidates, respectively. The CLE motifs of the closest *AtCLE* genes were shown below the Weblogos.
